# Tough Cortical Bone‐Inspired Tubular Architected Cement‐Based Material with Disorder

**DOI:** 10.1002/adma.202313904

**Published:** 2024-09-10

**Authors:** Shashank Gupta, Reza Moini

**Affiliations:** ^1^ Department of Civil and Environmental Engineering Princeton University Princeton NJ 08544 USA

**Keywords:** cement‐based material, cortical bone, fracture toughness, order parameters, statistical mechanics, tubular architecture

## Abstract

Cortical bone is a tough biological material composed of tube‐like osteons embedded in the organic matrix surrounded by weak interfaces known as cement lines. The cement lines provide a microstructurally preferable crack path, hence triggering in‐plane crack deflection around osteons due to cement line‐crack interaction. Inspired by this toughening mechanism and facilitated by a hybrid (3D‐printing/casting) process, the study engineers architected tubular cement‐based materials with the stepwise cracking toughening mechanism, that enables a non‐brittle fracture. Using experimental and theoretical approaches, the study demonstrates the competition between tube size and shape on stress intensity factor from which engineering stepwise cracking can emerge. Two competing mechanisms, both positively and negatively affected by the growing tube size, arise to significantly enhance the overall fracture toughness by up to 5.6‐fold compared to the monolithic brittle counterpart without sacrificing the specific strength. This is enabled by crack‐tube interaction and engineering the tube size, shape, and orientation, which promotes rising resistance‐curves (R‐curve). “*Disorder*” curves and statistical mechanics parameters are proposed for the first time to quantitatively characterize the degree of disorder for describing the representation of the architected arrangement of materials in lieu of otherwise inadequate “*periodicity*” classification and misperceived disorder parameters (perturbation and Voronoi tessellation methods).

## Graphical abstract

Disordered tubular architected cement‐based materials inspired by cortical bone exhibit stepwise cracking toughening mechanism, preventing brittle fracture in otherwise brittle material. Competing effects of tube size, shape, and orientation demonstrate a utility for achieving up to 5.6‐fold‐higher fracture toughness. Two order parameters from statistical mechanics provide pathway to quantify disorder in architected arrangements as described by Reza Moini and co‐worker.

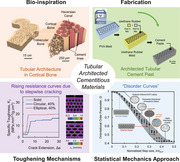

## 1 Introduction

Natural materials demonstrate an exceptional combination of two often competing mechanical properties, fracture toughness and strength, by assembling modest constituents into complex arrangements and hierarchical architectures.^[^ ^1^, ^2^, ^3^, ^4^, ^5^, ^6^, ^7^, ^8^, ^9^ ^]^ Numerous biological materials, composed of large amounts of highly brittle minerals such as aragonite^[^ ^10^, ^11^ ^]^ and hydroxyapatite^[^ ^12^, ^13^ ^]^ achieve significantly higher fracture toughness by exploiting the purposeful arrangements, also known as materials’ architecture, such as helical,^[^ ^12^, ^13^, ^14^ ^]^ gradient,^[^ ^15^, ^16^ ^]^ layered,^[^ ^17^, ^18^ ^]^ suture,^[^ ^19^, ^20^ ^]^ and tubular.^[^ ^21^, ^22^ ^]^ High fracture toughness in these motifs is enabled by unique toughening mechanisms such as crack twisting in helical,^[^ ^23^ ^]^ structural reorientation in gradient,^[^ ^24^ ^]^ crack deflection along the interface in layered,^[^ ^25^ ^]^ and interlocking in the suture^[^ ^26^ ^]^ architected materials.

On the contrary to natural counterparts, fracture toughness and strength, are often two competing mechanical properties in engineering materials.^[^ ^27^, ^28^, ^29^ ^]^ For instance, technical and non‐technical (e.g., concrete) ceramics can exhibit high strength but are limited by their low fracture toughness, relative to other classes of materials such as metals.^[^ ^30^ ^]^ Concrete is the most commonly used human‐made commodity in the world^[^ ^31^ ^]^ and suffers from intrinsically low fracture toughness owing to the limited toughening mechanisms.^[^ ^32^, ^33^, ^34^, ^35^, ^36^, ^37^, ^38^, ^39^ ^]^ Consequently, this translates to the low fracture toughness under tension in cement‐based materials including concrete,^[^ ^32^, ^33^, ^36^ ^]^ mortar,^[^ ^35^, ^37^, ^38^ ^]^ and cement paste.^[^ ^34^, ^37^, ^38^, ^39^ ^]^ More specifically, the low fracture toughness of the binding phase of the concrete matrix, the cement paste, is due to the limited ability to dissipate energy during the crack initiation and propagation throughout the microstructure.^[^ ^40^ ^]^ Here, we present a viable mesoscale approach to engineer a toughening mechanism throughout the crack growth in the materials, by harnessing tubular voids that can promote crack‐tube interaction and lead to stepwise cracking.

**Figure 1:**
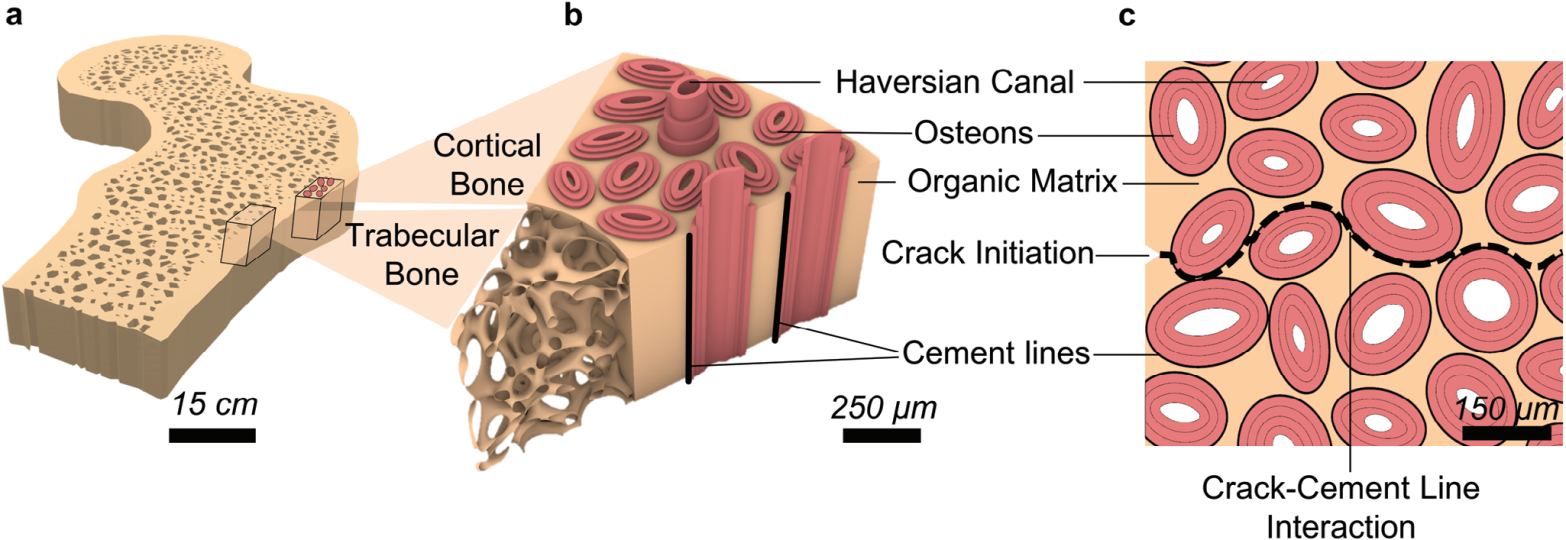
Architecture of cortical bone. a) Schematic cross‐section of human femur bone illustrates the dense outer cortical bone and porous inner trabecular bone. b) Cortical bone architecture depicts the presence of weak cement lines surrounding tubular osteons, which leads to c) crack‐cement line interaction, providing a path for in‐plane crack‐deflection from a straight brittle fracture as the toughening mechanism.

Tubular architecture has been exploited in several biological materials, such as human teeth,^[^ ^41^ ^]^ human cortical bone,^[^ ^42^ ^]^ horse hooves,^[^ ^43^ ^]^ bamboo,^[^ ^44^ ^]^ and ram horns,^[^ ^45^ ^]^ to promote fracture resistance while serving metabolic functions.^[^ ^25^, ^46^, ^47^ ^]^ The human femur bone, in particular, is composed of an outer cortical region^[^ ^42^ ^]^ and an inner trabecular region^[^ ^48^ ^]^ (**Figure** **1**a). The cortical region consists of dense lamellated tubular osteons,^[^ ^3^ ^]^ whereas the inner trabecular region is porous and contains an interspersed and interconnected network of plates and rods^[^ ^49^ ^]^ (Figure 1b). Cortical bone demonstrates exceptional fracture toughness due to its hierarchical structure that leads to resistance in the initiation and propagation of cracks through various intrinsic and extrinsic multi‐scale toughening mechanisms.^[^ ^50^ ^]^ These mechanisms include molecular uncoiling and fibrillar sliding at the nano‐scale,^[^ ^50^ ^]^ micro‐cracking and sacrificial bonds at the sub‐micron scale,^[^ ^50^, ^51^ ^]^ as well as collagen‐fiber bridging, uncracked‐ligament bridging, crack deflection, and crack twisting at the micron scale.^[^ ^3^, ^7^, ^50^, ^51^, ^52^ ^]^ Notably, we focus on crack deflection, triggered due to micro‐scale osteons, also known as the Haversian system.^[^ ^3^, ^7^, ^50^, ^51^, ^52^ ^]^ The osteons in the cortical bone are embedded in the organic matrix encapsulating Haversian canals for metabolic function and are surrounded by a particular weak interface known as cement lines^[^ ^52^ ^]^ (Figure 1b,c). The cement lines have a specific mechanical characteristic as they have 10‐fold lower tensile strength compared to the osteons they surround.^[^ ^53^ ^]^ The typical elliptical cement lines, due to the weaker tensile strength, provide microstructurally preferred pathways for interaction with a propagating crack.^[^ ^52^ ^]^ Hence, cement lines trigger unique toughening mechanisms in the cortical bone that involve in‐plane crack deviation from a straight path and crack deflection around osteons, as schematically illustrated in Figure 1c.^[^ ^54^, ^55^ ^]^ This type of crack deflection prevents a brittle fracture^[^ ^51^ ^]^ and is the key toughening mechanism investigated in this study to inform an initial design of tough engineering counterparts. In other words, the bio‐inspired approach in this work has focused on a one‐mechanism‐at‐a‐time strategy in learning the principles of toughening mechanism in cortical bone (crack deflection and crack‐tube interaction) for engineering additional toughening mechanisms in engineered counterparts.

More specifically, here the particular interaction between the crack and the tubular geometry surrounding osteons found in cortical bone at the micro‐scale is exploited for engineering a toughening mechanism in brittle hardened cement paste material. This is enabled by circular and elliptical hollow tubular architecture at the meso‐scale based on cortical bone.^[^ ^37^ ^]^ More specifically, the design of the tubular architected materials is inspired by the consideration of the volume fraction^[^ ^56^ ^]^ (45–65%) and shape (aspect ratio, *e*)^[^ ^57^ ^]^ (nearly circular to elliptical, with an aspect ratio of *e* = 1.02–2.62) of osteons (surrounded by cement line) in cortical bone. A periodic but disordered arrangement is considered in this study for the design of circular and elliptical tubes. The *disorder* is proposed as a more suitable metric, in contrast to periodicity (as a binary metric), to quantify the arrangement as a spectrum from a statistical mechanics perspective using radial distribution function, *g*_2_(*r*), and two order parameters (Translational (*T)* and Orientational (*Q*) order parameters). We hypothesize that engineering the crack‐tube interaction toughening mechanism through designing tubular architected brittle cement‐based material may lead to enhanced fracture toughness in an otherwise purely brittle material.^[^ ^37^ ^]^

While maintaining volume fraction and aspect ratio design parameters based on the Haversian system, it should be noted that our bio‐inspired design approach does not attempt to mimic the hierarchical arrangements of osteons observed in cortical bone. Rather, here we draw upon one of the underlying toughening mechanisms of crack‐cement line interaction as an inspiration to expand upon. This approach to bio‐inspired design facilitates understanding and engineering of a specific toughening mechanism, in contrast to the complexities that might arise from attempting a full emulation of the hierarchical structures of cortical bone.

To date, a few studies have only explored the design of composites inspired by cortical bone architecture by making use of inclusions (in the voids) to improve energy absorption (toughness),^[^ ^58^ ^]^ fracture toughness,^[^ ^59^ ^]^ and flexural strength.^[^ ^59^, ^60^ ^]^ For instance, bone‐inspired fibers‐reinforced composites have been fabricated to enhance fracture toughness, by enabling crack deflection toward the inclusion boundaries, compared to solid laminated counterparts without inclusions. However, mere crack deflection is prevented at a small void size, and if present at a large void size, can be macroscopically undermined due to insignificant contribution to the stress required to advance the crack at the notch residing behind a void.^[^ ^61^ ^]^ The bone‐inspired composites exhibited limited improvement of up to 26% in fracture toughness and 30% in flexural strength compared to conventionally laminated counterparts^[^ ^59^, ^60^ ^]^ or 6.5 fold higher energy absorption (toughness) and 3.7 folds lower strength compared to solid counterparts by making use of stiff inclusions in a soft matrix.^[^ ^58^ ^]^ In other studies, cast fiber‐reinforced composites with tubular voids have been proposed to engineer auxeticity.^[^ ^62^, ^63^ ^]^ Another study investigates tubular voids with circular designs in ductile material to enhance fracture toughness.^[^ ^64^ ^]^ This research demonstrates the improvement in fracture toughness in ductile material for a notch with a blunt tip located *on* the tube that competes with the increasing circular tube size for higher fracture toughness. This approach couples the effect of the tube size on the notch tip (crack blunting) with the effect of the surrounding tubes on the (blunt) notch (hole–hole). In contrast, here we examine the effect of the circular and elliptical tube(s) *ahead* of the crack tip on a sharp notch, followed by the investigation of crack‐tube interaction that enables stepwise cracking, where we hypothesize the increasing tube size impacts fracture toughness both negatively (due to increased stress intensity factor) and positively (due to enabling stepwise cracking). Several bio‐inspired strategies observed at the micron scale in cortical bone have been adapted in human‐made materials at the millimeter scale.^[^ ^58^, ^59^, ^60^ ^]^ Moreover, while limited studies have explored tubular geometry in composite and ductile material, the toughening mechanisms in brittle material counterparts have not been readily reported.

Contrary to the previous works where voids and inclusions are used to enhance crack deflection, we harness a new strategy to exploit the geometry (size, shape or aspect ratio) of tubes in tubular architecture itself as purposeful defects to engineer a new toughening mechanism and enhance fracture characteristics. Engineering the critical stress intensity factor (fracture toughness) through understanding the interactions of crack with the tube assists in allowing for a macroscopically effective stepwise cracking and highly non‐brittle failure in an otherwise brittle material.

## 2 Results

**Figure 2:**
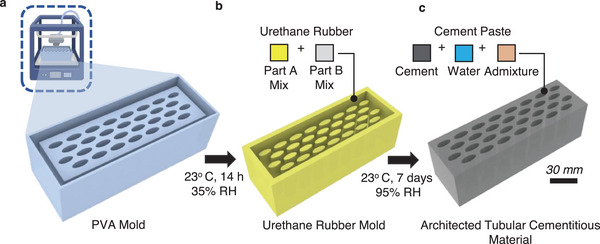
Fabrication of tubular architected cement‐based materials. a) 3D‐printing of a PVA template mold, followed by b) generation of a negative urethane rubber mold by dissolving the PVA template, and c) casting tubular architected cement‐based material.

To fabricate the tubular architected cement‐based materials, we use a hybrid 3D‐printing and casting approach. Firstly, a tubular template mold is additively manufactured using 3D‐printing with polyvinyl alcohol (PVA) (**Figure** **2**a). Furthermore, the negative of the template is fabricated by pouring two‐part urethane rubber into the PVA template, which is subsequently dissolved to create a urethane silicon mold (Figure 2b). Tubular architected cement‐based materials are cast (Figure 2c) into this silicon mold. Additional details of the fabrication process are described in the Methods.

To study the effect of tube ellipticity on the mechanical response of the material, the aspect ratios of the tubes vary from 1.0 to 3.0 (*e* = 1, 2, 2.5, 3) based on the aspect ratio of the osteons in cortical bone (1.02 < *e* < 2.62), where *e* = 1 represent a circle. To understand the presence of a potential relation between the spatial arrangement of the tubes and crack path, in the circular and elliptical designs, the degree of *disorder* was characterized and quantified by using two scalar quantities (*T*, *Q*) and radial distribution functions using a statistical mechanics approach in comparison to the theoretical perfectly random (ideal gas) and a perfectly ordered (Hexagonal closed packing (HCP) crystal) in two dimensions (Figure S1, Supporting information). It was found that although the circular and elliptical designs are periodic, they are not perfectly ordered, and thus have translational (*T*) and orientational (*Q*) order parameters below 1 (1 represents a perfect crystal) (Figure S2, Supporting information).

**Figure 3:**
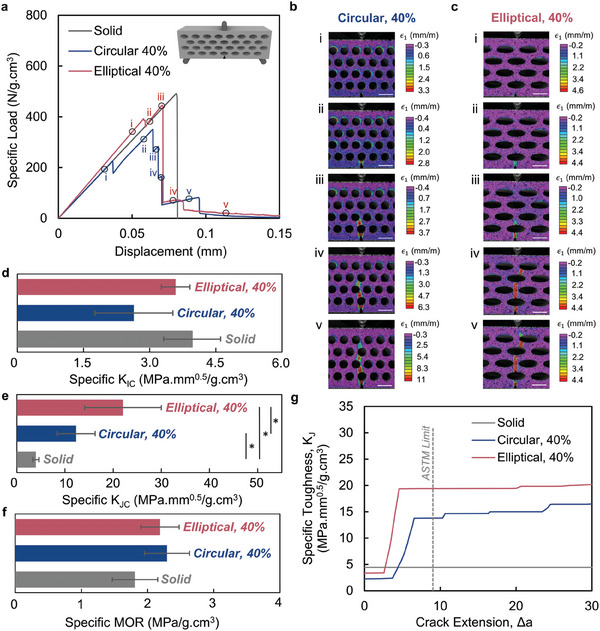
Mechanical response of circular and elliptical tubular architected cement‐based materials compared to the solid counterparts. a) Specific load versus displacement curves from the SENB test with the corresponding digital image correlation (DIC) analysis based on maximum principal strain (ɛ_1_) of b) Circular, 40%, and c) Elliptical, 40% designs. d,e) Comparison of the specific crack initiation fracture toughness (K_IC_) and crack propagation fracture toughness (K_JC_) of tubular and solid materials. f) Specific modulus of rupture (MOR) of architected and monolithic solid materials obtained from the three‐point bending test. g) Specific toughness (calculated using J‐integral) versus crack extension (Δa) demonstrating rising R‐curves for architected tubular materials compared with the constant fracture toughness of monolithic solid counterpart. All scale bars are 10 mm. Data is shown as mean ± SD. “*” depicts *p* < 0.05 which indicates the statistically significant difference between the samples (at the ends of the solid line below a “*”). *p*‐value is obtained from the F‐test and T‐test.

We characterize the fracture response and modulus of rupture (MOR) of the tubular architected cement‐based materials using single‐edge notched bending (SENB) and three‐point bending test, respectively, for various designs (i.e., circular and elliptical) and porosities (*φ* = 20%, 30%, 40%, and 50%) in comparison with the reference solid counterparts fabricated with the same material composition (**Figure** **3**; Figures S5 and S6, Supporting Information). More specifically, Figure 3 represents specific load versus displacement plots obtained from the SENB test, specific crack initiation and crack propagation fracture toughness, R‐curve, and specific MOR for the case of circular (*φ* = 40%) as well as elliptical (*φ* = 40%, *e* = 2.5) designs. Circular, 40% and Elliptical, 40% (*e* = 2.5) are chosen for conciseness and detailed discussion as they exhibit the most promising fracture response among the respective circular and elliptical designs of the tubular architected materials.

In comparison to the conventional single brittle failure in the solid counterparts (shown in grey), the specific‐load versus displacement response of tubular architected material obtained from the SENB test (Figure 3a), highlights a unique globally non‐brittle and non‐abrupt fracture behavior (shown in red and blue). The global non‐brittle behavior is characterized by a postponed abrupt failure after the initial peak load provided by the circular and elliptical tubular architecture. Unlike the catastrophic failure at the first peak load in solid brittle materials, the tubular architected designs postpone the abrupt failure and exhibit multiple load drop and increase steps hence allowing for an overall hardening and softening behaviors after the first peak.

Analysis of the fracture profiles for Circular, 40% (Figure 3b; Movie S1, Supporting Information) and Elliptical, 40% (Figure 3c; Movie S2, Supporting Information), obtained through digital image correlation (DIC), reveals more insight into the relationship between the multi‐step attributes of the load versus displacement curve (Figure 3a) and the crack path (Figure 3b,c). The first load drop in the response of Circular, 40% and Elliptical, 40% (Figure 3a) is shown in blue and red, respectively, and corresponds to crack initiation from the sharp notch to the tube in the bottom‐most row in these two architectures (Figure 3b(i,ii),c(i,ii)). This is followed by a common increase in the load in both the elliptical and circular cases (Figure 3a) and indicates the reinitiation of the crack from the first tube ahead of the initial notch and propagation toward the subsequent row of tubes, respectively (Figure 3b(ii,iii),c(ii,iii)). The subsequent load drops in the load‐displacement response (Figure 3) continue to correspond to the additional interaction of the crack with the tubes ahead of the crack tip in both types of designs (Figure 3b(iii–v),c(iii–v)). This unique behavior underscores the remarkable ability to pin the crack to the tube that acts to stabilize the overall crack propagation by promoting crack‐tube interaction. This phenomenon gives rise to the overall non‐brittle non‐abrupt response in the circular and elliptical tubular architected cement‐based materials compared to the cast counterparts.

The fracture response of tubular architected materials and the solid counterparts is presented in terms of specific crack initiation fracture toughness (K_IC_) and specific crack propagation fracture toughness (K_JC_), as illustrated in Figure 3d,e, respectively. The Circular, 40% and Elliptical, 40% display the specific K_IC_ of 2.64  ± 0.88 MPa mm^0.5^/g cm^3^ and 3.59  ± 0.32 MPa mm^0.5^/g cm^3^ which is statistically similar to the solid cement‐based material, 3.96  ± 0.64 MPa mm^0.5^/g cm^3^ (in the confidence level of 95%). A similar K_IC_ normalized with respect to density indicates that the presence of tubular architecture does not lead to any significant loss of resistance to cracking.

In addition to specific K_IC_, the specific crack propagation fracture toughness (K_JC_) can be characterized to capture the resistance to cracking in cases where the material does not abruptly fail in a brittle manner upon crack initiation. The Circular, 40% and Elliptical, 40% exhibit the specific K_JC_ of 12.30  ± 3.96 MPa mm^0.5^/g cm^3^ and 22.04  ± 7.96 MPa mm^0.5^/g cm^3^ corresponding to the ultimate resistance that takes place at the point of complete failure of the notched specimens. These values of K_JC_ for circular and elliptical architected materials are significantly higher than those of solid counterparts by 3.1 and 5.6 folds, respectively. Furthermore, the ASTM limit of K_JC_ of Elliptical, 40% is 20.48  ± 8.71 MPa mm^0.5^/g cm^3^ is significantly higher than that of solid material. Circular, 40% demonstrates the ASTM limit of K_JC_ as 8.92  ± 5.28 MPa mm^0.5^/g cm^3,^ which is statistically similar to its solid counterpart.

In addition, the specific MOR of the Circular, 40%, and Elliptical, 40% are 2.22  ± 0.33 MPa/g cm^3^ and 2.12  ± 0.28 MPa/g cm^3^, respectively, which are statistically similar to that of the solid material (1.76  ± 0.33 MPa/g cm^3^) as shown in Figure 3f. The findings show that engineering the tubular architecture to enhance the specific fracture toughness of the brittle cement‐based material can be achieved while maintaining its specific MOR (i.e., the presence of the tubes has not led to additional loss of strength). The results assert the ability to overcome the often mutually exclusive nature of these two properties in brittle materials by developing a stable crack propagation through leveraging tubular architecture.

The R‐curve plot captures the specific fracture toughness as a function of crack growth (Δ*a*) in tubular architected material in comparison to the solid material (Figure 3g). Therefore, the elegance of the R‐curve is in demonstrating the entire fracture process and the crack initiation, stepwise cracking through interaction with the circular and elliptical tubes, and overall crack propagation path until ultimate failure. In comparison to the brittle solid cement‐based materials that typically demonstrate a brittle failure upon crack initiation (only a constant K_IC_) as illustrated with a constant value on the Y axis with a gray line in Figure 3g, architected tubular material of the same composition represent a drastically dissimilar resistance curve. The R‐curve demonstrates that the specific fracture toughness of tubular architected material increases in a stepwise manner where an iterative series of rapid crack extensions (Δ*a*) on the x‐axis are followed by the increase in the specific fracture toughness on the y‐axis. These stepwise crack extensions from the fracture analysis used for the R‐curve (based on the load drop) are compared with the experimentally observed crack extensions (from the DIC) and represent a great agreement (Figure S7, Supporting Information). In other words, this stepwise rise in the R‐curve is directly correlated with the hardening and softening behavior observed as the load increase and load drop in the load‐displacement plot in Figure 3a at the events of crack initiation from the notch and the subsequent reinitiation from the tubes (Figure S7, Supporting Information). In contrast to the flat resistance curve in cast brittle cement‐based tubular architected counterparts of the same constituents provide unique rising R‐curves and higher fracture toughness.

## 3 Discussion

The interaction of the crack with the tube and the subsequent stepwise cracking is the toughening mechanism for tubular architected material made up of brittle constituent. Stepwise cracking involves a mechanism in which the tube first arrests the crack, followed by the crack reinitiation from the tube. Corroboration of crack‐tube interaction using DIC and the fracture toughness calculations from the load‐displacement provides evidence for such a characteristic of stepwise cracking (Figure 3a–c; Figure S7, Supporting Information). For instance, the five and four steps in crack extension based on the R‐curve very well match the five and four steps in the observed crack for the Circular 40% and Elliptical 40% tubular architected material, respectively (Figure S7, Supporting Information). This continued and stepped crack propagation ultimately provides the rise in the R‐curve, additional stepwise crack extension (Figure S7a,c, Supporting Information), and higher fracture toughness in tubular architected cement‐based materials.

**Figure 4:**
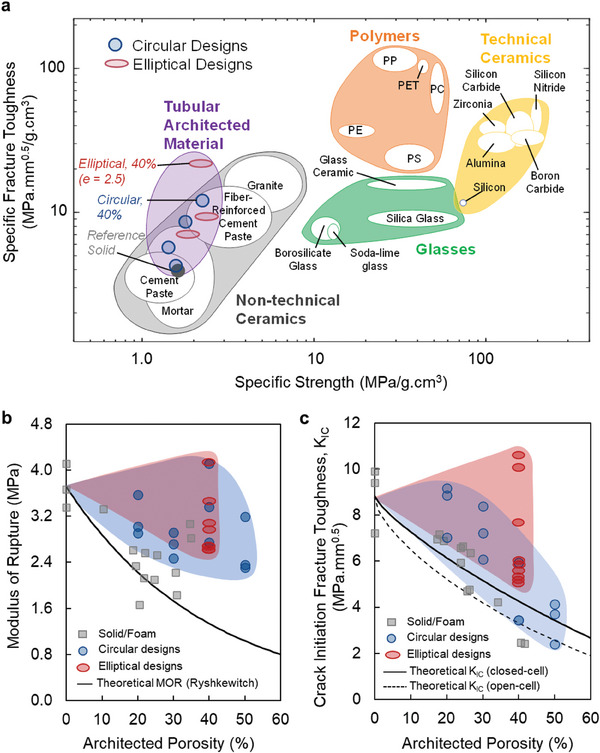
Ashby plot and modulus of rupture of architected material. a) Ashby plot displaying specific fracture toughness versus specific strength, featuring tubular architected cement‐based materials in comparison to our solid cement paste as well as the survey of the literature for cement paste,^[^ ^35^, ^37^, ^65^, ^66^, ^67^, ^68^, ^69^, ^70^ ^]^ fiber‐reinforced cement paste,^[^ ^71^, ^72^, ^73^, ^74^, ^75^ ^]^ and mortar^[^ ^35^, ^37^, ^67^, ^73^ ^]^ alongside various engineering materials, where the most promising architected materials exhibit higher specific fracture toughness than solid cast, and even mortar, and fiber‐reinforced cement paste counterparts, all while maintaining their specific strength. b,c) MOR and K_IC_ of tubular architected cement‐based materials with varying porosity (*φ* = 20%, 30%, 40%, and 50%) and aspect ratio (*e* = 1, 2, 2.5, and 3) compared to reference solid cement‐based material and theoretical MOR and K_IC_ (for open‐cell and closed‐cell material) obtained using the Ryshkewitch relation^[^ ^76^ ^]^ and geometric model, respectively.^[^ ^77^ ^]^

The Ashby plot (**Figure** **4**a) demonstrates the comparison of the specific fracture toughness versus specific strength of all investigated designs of tubular architected against the solid cement paste and fiber‐reinforced cement paste materials and mortar, as well as other engineered materials such as glass, polymers, and technical ceramics.^[^ ^78^ ^]^ The specific fracture toughness and specific flexural strength of solid reference cast closely lie within the (upper limit) range of values obtained from the literature. The circular designs with smaller porosity (*φ* = 20% and 30%) exhibit slightly higher specific fracture toughness compared to solid cement‐based material. However, the circular and elliptical designs with higher porosity (*φ* = 40% and 50%) demonstrate 3 and 2 folds higher fracture toughness, respectively, than the solid cement‐based material representing the conventional properties of cement paste, placing them in the same toughness range as the fiber‐reinforced cement paste. Specifically, the most effective design of tubular architecture, the Elliptical, 40% shows a specific fracture toughness of 22.04  ± 7.96 MPa.mm^0.5^/g.cm^3,^ which exceeds the specific toughness of the cementitious materials found in the literature for conventional cement paste (3.89  ± 1.5 MPa mm^0.5^/g cm^3^),^[^ ^35^, ^37^, ^65^, ^66^, ^67^, ^68^, ^69^, ^70^ ^]^ mortar (4.94  ± 3.5 MPa mm^0.5^/g cm^3^),^[^ ^35^, ^37^, ^67^, ^73^ ^]^ and fiber‐reinforced cement paste (10.12  ± 4.2 MPa mm^0.5^/g cm^3^).^[^ ^71^, ^72^, ^73^, ^74^, ^75^ ^]^

On the other hand, compared to the theoretical MOR obtained using the Ryshkewitch relation^[^ ^76^ ^]^ and reference solid foam cast material, the circular and elliptical tubular architected materials display a relatively higher MOR (Figure 4b; Figures S5e and S6e, Supporting Information). Similarly, we have compared the crack initiation fracture toughness (K_IC_) of tubular architected materials alongside that of foam cement and the theoretical K_IC_ values for open‐cell and closed‐cell materials (where the cell represents the pore phase in a geometrically conceptualized model^[^ ^77^ ^]^), as shown in Figure 4c. Tubular architected materials with circular designs generally outperform the solid foam in terms of K_IC_ at the examined range of (architected and entrained) porosity. Additionally, the circular architected materials exhibit higher K_IC_ at lower porosities (10–30%) when compared to the theoretical K_IC_ of closed cell counterparts^[^ ^77^ ^]^ and at higher porosities (40–50%), converge toward the theoretical value for K_IC_ for open‐cell materials.^[^ ^77^ ^]^ Moreover, the elliptical tubular architected materials examined here, surpass solid foam materials, and both closed‐cell and open‐cell theoretical K_IC_^[^ ^77^ ^]^ at comparable porosities.

Investigation of a broader range of the porosity in the circular architected materials and comparison against cast counterpart demonstrates an additional insight about the optimality of the “tubular porosity” of *φ* = 40% case compared to other cases. The outperformance of the *φ* = 40% case in terms of fracture toughness is enabled by the effectiveness of the stepwise cracking toughening mechanism in this case. By conducting a theoretical analysis of the stress intensity factor, K_I_ at the notch, due to the presence of a single tube ahead of the notch (Figures S9–S11, Supporting Information), the underlying phenomena about the relation between the stress field and stress intensity factor can be elucidated. It follows that as the tubular porosity, *φ*, increases by increasing the radius of the tube, the stress intensity factor at the notch due to the presence of the hollow tube increases leading to a lower load to initiate a crack (Figure S9c,d, Supporting Information). The increase in stress intensity factor results in the reduction in the stress and corresponding value of the peak load required for initiation of the crack from the notch tip as the tubular porosity increases ahead of the tip (Figure S9e,f, Supporting Information). Thus, at the largest (manufacturable) tube size *φ =* 50%, the lowest fracture toughness is expected, given the relationship between load and fracture toughness. At the smallest tube size at *φ =* 20%, the highest load is expected. However, the other key to enhanced fracture toughness is the ability to promote the crack‐tube interaction, which gives rise to the cracking and rising R‐curves.

These two mechanisms compete, as evident from the load‐displacement plot. As the tubular porosity increases, although the fracture toughness should decrease, the larger tubes enable the crack to interact with the tube and provide crack arrest, which provides a pathway for more stable crack propagation and a rising R‐curve. Although at large tube sizes *φ =* 50%, the stepwise cracking remains and provides stepwise crack extension as seen in the R‐curves and load versus CMOD plot (Figure 3g; Figures S5b and S8a, Supporting Information), its effect diminishes as the critical load to initiate the crack significantly decreases with increasing tube size. On the other hand, at the smallest tube size *φ =* 20%, the spare distribution of the voids due to their small size does not promote crack‐tube interaction, though it may provide higher peak load and fracture toughness at the initiation. This gives rise to an optimal tubular porosity of *φ =* 40% (Figure S5d, Supporting Information), which was extended to an elliptical design with varied aspect ratios.

In terms of aspect ratio, the best performance of *e* = 2.5 in terms of fracture toughness is attributed to the competing stepwise cracking toughening mechanism and *e*. For a single tube interacting with a crack, an increase in *e* leads to a decrease in the K_I_ (Figure S9d, Supporting Information), which in turn necessitates an increase in the peak load required to initiate the crack (Figure S9e,f, Supporting Information). This finding suggests that the case of *e* = 3 is expected to have the highest fracture toughness, whereas the case of *e* = 2 is expected to have the lowest fracture toughness. However, fracture toughness also depends on the growth in the R‐curve, which is a result of the stepwise cracking toughening mechanism. Thus, as stepwise cracking competes with *e* for the peak required for crack initiation, the lower aspect ratio (*e* = 2 and *e* = 2.5) promotes stepwise cracking, in contrast to *e* = 3 which does not exhibit stepwise cracking, as shown by R‐curve and load versus CMOD plots (Figure 3g; Figures S6b and S8b, Supporting Information). Considering the peak load and the stepwise cracking toughening mechanism, *e* = 2.5 demonstrates the highest fracture toughness compared to all tubular designs (Figure 3e; Figure S6d, Supporting Information).

While this study focuses on the effect of tube geometry, shape, and orientation on stress intensity factor and fracture toughness among studied systems in isolation, a broader distribution and combination of these geometric features can be explored. The experimental and theoretical insight from the underlying geometry‐stress intensity factor relationship and crack‐tube interactions in this study can be used in the future design to trigger stepwise cracking while prohibiting high stress intensity factor (low peak load) at each crack initiation step among various distributions or combinations of geometric features (e.g., in isolation or homogenized).

Here, we engineer the toughening mechanism of stepwise cracking, which includes crack arrest and crack reinitiation, in cement‐based tubular architected material with an inherently brittle constituent inspired by the crack‐cement line interaction in cortical bone. Engineering tubes as defects ahead of the crack in cement‐based materials can help generate unique toughening mechanisms inspired by defect‐crack interaction in cortical bone. It should be noted that the stepwise toughening mechanisms emerge when the volume fraction of tubular voids reaches 40 – 50% (of both circular and elliptical cases), a range that aligns closely with the 45 – 60% volume fraction of osteons in cortical bone.^[^ ^56^ ^]^ Additionally, the most promising case of elliptical design with 40% porosity and aspect ratio *e* = 2.5, is also within the matching range of the osteons' volume fraction and falls in the aspect ratio range of osteons in cortical bone (*e* = 1.02 to 2.62).^[^ ^57^ ^]^

Further studies can investigate the arrangement of the tubes ahead of the crack. Using a statistical mechanics approach, further understanding of the seemingly periodic versus non‐periodic defects such as tubular porosity can be achieved by translating periodicity to “order parameters”. This approach can help formalize a quantifiable metric into the design of materials arrangement (solid or pore) and assist in performing inverse design of tubular porosity. From a statistical mechanics perspective, two limits of random (ideal gas) and perfectly ordered (Face‐centered cubic (FCC) crystal) can be constructed with a “*disorder*” spectrum in between.^[^ ^79^, ^80^ ^]^ From a design of architected materials perspective, a wide range of disorder arrangements (of tubular porosity or any other architecture and phase), bound by the random and ordered limits, can be conceptualized and mechanically probed in the context of fracture mechanics. In this work, a rigorous approach to characterize the arrangement of architected material is proposed for the first time with the preposition to move away from inadequate periodicity concepts and toward the mathematically conceivable representation of the materials using disorder (i.e., degree of disorder) in the field of architected materials. As such, we propose to capture “disorder” using radial distribution function (Figures S1,S2, and S4, Supporting Information) and more quantitatively, by related translational order parameter, *T* (See Equation S12, Supporting Information), and orientational parameters, *Q* (See Equation S13, Supporting Information) in this endeavor.

The random system is the lower bound for *T* and *Q*, and the perfect FCC crystal (in 3D) or HCP crystal (in 2D) is the upper bound for *T* and *Q*, based on statistical mechanics.^[^ ^79^, ^80^ ^]^ In this study, given the 2D nature of the tubular design, we suffice to investigate the order parameters for the proposed materials in 2D systems, leading to a range from an HCP lattice to random distributions.

**Figure 5:**
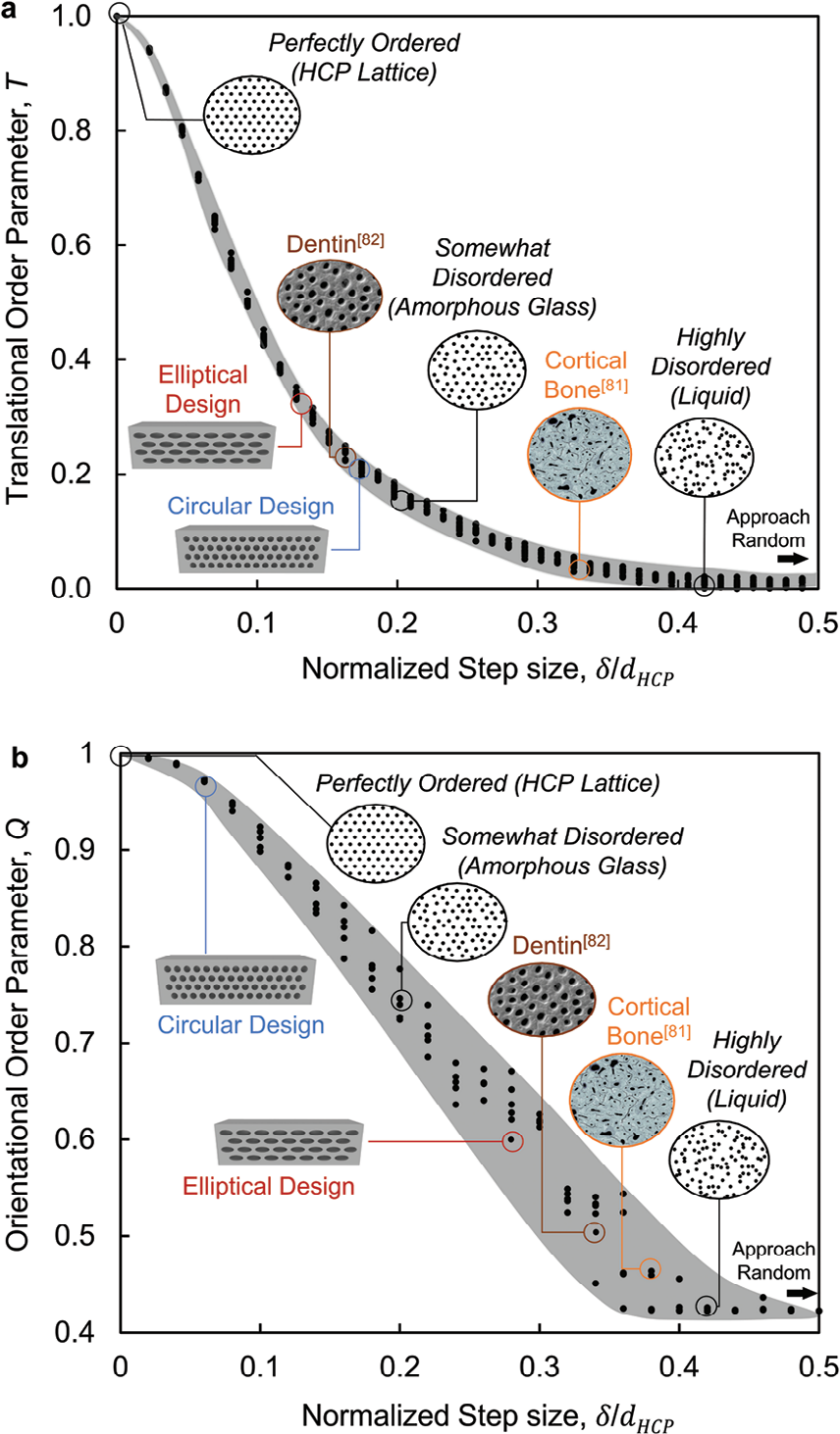
Disorder curves representing order parameters (T, Q) versus Normalize step size based on a statistical mechanics approach, imposed on them the order parameter values of natural and engineered tubular architected materials. a) Translational order parameter (*T*) and b) orientational order parameter (*Q*) with varying step size (δ/*d_HCP_*) for 2D – distributions, ranging from perfectly ordered, to disordered (“somewhat” and “highly” corresponding to amorphous glass and liquid), ultimately approaching random. The order parameters were independently computed based on the distribution of centroids of tubes in tubular architected material (Circular and Elliptical arrangement) along with the distribution of the centroids of osteons in cortical bone adapted from^[^ ^81^ ^]^ and tubules adapted from^[^ ^82^ ^]^ in Dentin are presented on *T* versus δ/*d_HCP_* and *Q* versus δ/*d_HCP_* disorder curves. The step size (δ/*d_HCP_*) controls the degree of disorder in the system with δ  =  0 indicating the perfect lattice and δ  =  0.5*d_HCP_* as the highly disordered system. The variation in the *T* and *Q* values for the fixed value of step size (δ/*d_HCP_*) is statistical noise, which is generated due to the stochastic nature of the Metropolis algorithm used to generate the disordered pattern. More specifically, the random sampling of displacement of particles within the maximum allowable step size (δ) in the Metropolis algorithm (Figure S3, Supporting Information) generates this statistical noise. The gray strips in *T* versus δ/*d_HCP_* and *Q* versus δ/*d_HCP_* disorder curves encompass the statistical noise generated from 10 repetitions.

Thus, within the 2D systems, we examine the translational order parameter (*T*) and the orientational order parameter (*Q* $=Q_{6}/Q_{6}^{HCP}$), for these limits leading to range from 1 to 0 for *T* and from 1 (HCP) to 0.42 for *Q* (equivalent to 0.74 to 0.31 for *Q_6_*), respectively. This variation in *T* and *Q* parameters corresponds to a range from “perfectly ordered” to “highly disordered” systems (where *Q* approaches 0 in 3D but not in 2D) and is illustrated in **Figure** **5**a,b, respectively. This range is broad and encapsulates the degree of disorder as it exists in the arrangement of both tubular cases probed in this study as well as the natural materials. The tubular architecture in nature exhibits a high degree of disorder, with tubules in Dentin showing *T* and *Q* parameter values as 0.22 and 0.51, respectively, whereas osteons in cortical bone display *T* and *Q* parameter values as 0.02 and 0.47, respectively.

The rigorous framework for capturing disorder using radial distribution function, *g*_2_(*r*) (Figure S4, Supporting Information) and *T* and *Q* order parameters, for a range of broad distributions from perfectly ordered to highly disordered (approaching random in 2D), were developed by considering a normalized step size (using metropolis algorithm – Figure S3a–d, Supporting Information). This leads to obtaining what we refer to here as “*disorder curves*” representing the *T* and *Q* as a function of step sizes δ, normalized by the first nearest neighbor distance for the HCP (*d_HCP_*) as shown in Figure 5a,b. It is worth noting that the order *T* can be quite sensitive to the statistical mechanics parameters used to calculate it (i.e., number of particles N, shell thickness Δ, and the number of shells N_s_). Thus, a thorough sensitivity analysis is conducted to allow for convergence of the T value to a plateau for each of these three parameters (See Figure S3e–i, Supporting Information).

Additionally, we use the void centroid to represent the arrangement of both natural and engineered materials to calculate the corresponding *T* and *Q* parameters values and impose them on the “disorder curves” (Figure 5a,b). The osteons in cortical bone and tubules in dentin exhibit the same degree of disorder as liquid (highly disordered) and glass (“somewhat disordered”), respectively, for both *T* (0.02 & 0.22) and *Q* (0.47 & 0.51) parameters.

The circular and elliptical tubular arrangements proposed in this work represent a translational order parameter, *T* of 0.21 and 0.30 (close to those dentin), and orientational parameters, *Q* of 0.97 and 0.60, respectively. This reveals that although the proposed circular and elliptical architected material appears periodic, the *T* parameter meaningfully represents a value corresponding to a “somewhat disordered” range of materials (Figure 5a).

In contrast, the proposed tubular architected material represents drastically different *Q* parameters between the circular and elliptical designs, indicating the utility of *Q* in the slight nuances of the design of the arrangement (Figure 5b). In addition, the *Q* parameter for the proposed circular architected materials represents an intuitively meaningful value of *Q* = 0.97, very close to a perfectly ordered HCP system (*Q* = 1), as depicted in Figure 5b. The *Q* parameter for the elliptical architected materials, on the other hand, represents a value of *Q* = 0.60, in the range between “somewhat disordered” and “highly disordered”, as illustrated in Figure 5b.

These findings highlight that neither “*periodicity*” is equivalent to “*ordered*” in a system, nor is “*non‐periodicity*” equivalent to “*disorder*”, highlighting a mechanically robust framework can provide better insight into the nature of a given design and the design‐performance relationship. Rather, the arrangement of phases in architected materials that can take any distribution other than perfect HCP lattice exhibit a certain degree of disorder considering either *T* or *Q* order parameters. Thus, the proposed set of order parameters can be powerful in tagging a given design with a quantifiable arrangement metric and probing for various mechanical and functional characteristics. The proposed algorithm is powerful in that it can be applied to any architected arrangement of materials beyond those imposed in Figure 5 here (natural and engineered tubular materials).

The findings highlight the effectiveness of *T* and *Q* in capturing the degree of disorder (e.g., heterogeneity) quantitatively in tubular and, more broadly, in the field of architected materials (Figures S1–S4, Supporting Information). More importantly, the proper application of statistical mechanics and “degree of disorder” can help suggest a better measure than the assessment via “periodicity”.^[^ ^83^, ^94^, ^95^ ^]^

Besides, the insufficient classification of periodic versus non‐periodic, there are major misconceptions in the definition of disorder as it pertains to its proper quantification. The lack of a fundamental method for the quantification of disorder has only exacerbated the notion of what is currently conceived as the disorder. This has prevented the field from decoupling the methods to “generate” disorder (and conceiving their associated parameters as “disorder parameters”) from “quantification” of disorder, which this study attempts to introduce for the first time.

**Figure 6:**
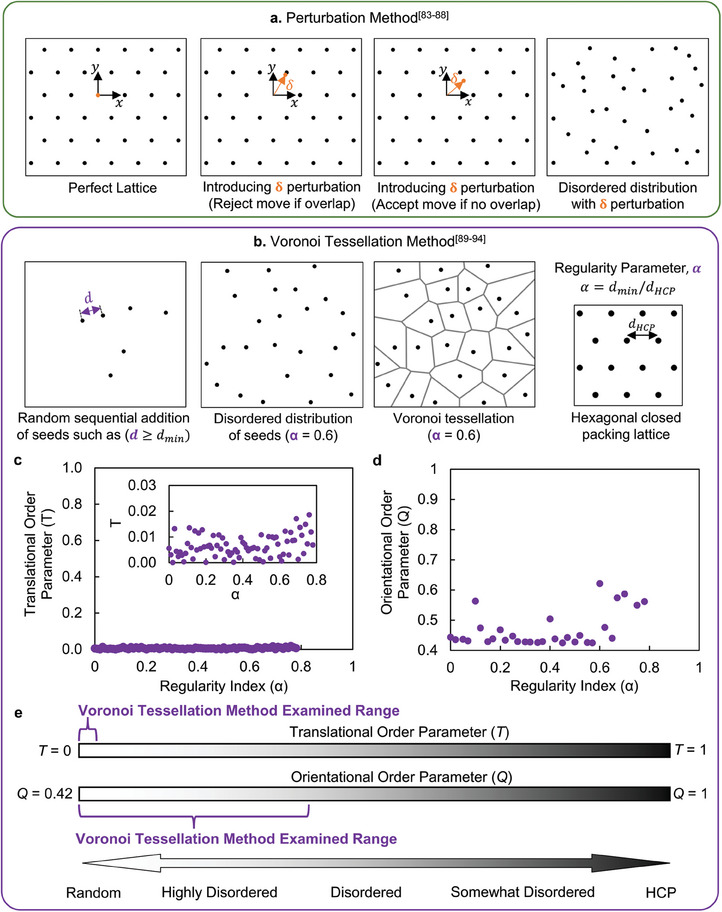
Comparison of the proposed statistical mechanics method to quantify disorder with two main existing methods in the field of architected materials to generate disturbance (perturbation) as “conceived” disorder. a) Perturbation method introduces small, random displacements, δ, to the positions of particles starting with a given perfect lattice (any periodic case) to set disturbances in the arrangement (i.e., increasing δ is to set higher disturbance from a perfect lattice), lacking quantification of disorder of the distribution itself as a dependent variable.^[^ ^83^, ^84^, ^85^, ^86^, ^87^, ^88^ ^]^ b) Voronoi tessellation method with random sequential addition to the arrangement using regularity parameter, α  =  *d_min_*/*d_HCP_* involving placement of seed points within a domain, maintaining a minimum distance, *d_min_* between each seed point, leading to only a random distribution.^[^ ^89^, ^90^, ^91^, ^92^, ^93^, ^94^ ^]^ c,d) Translational (*T*) and orientational (*Q*) order parameters quantified versus the regularity parameter, α  =  *d_min_*/*d_HCP_* demonstrating both the lack of quantification of disorder in Voronoi tessellation method as well as the limitation of the method (use of α) in producing sufficiently broad range of disorder of the distribution, and e) the limited range of the regularity parameter, α, in the conceived arrangement when examined against the proposed disorder parameters *T* and *Q* with a full range of the spectrum from random to HCP.

Several outstanding works in the field of architected materials have employed methods, such as the perturbation method^[^ ^83^, ^84^, ^85^, ^86^, ^87^, ^88^ ^]^ and the Voronoi tessellation method,^[^ ^89^, ^90^, ^91^, ^92^, ^93^, ^94^ ^]^ to generate disordered distributions. While these methods can be used to create disturbances (perturbation) in the designs, they are not suitable for characterizing the extent of the disorder. For instance, one of the key methods to generate disorder is the perturbation method (**Figure** **6**a) which has been widely used to conceive and introduce “disorder” in the perfect lattice. The method involves introducing small, random displacements, δ, (which is referred to as the “disorder parameter”^[^ ^84^ ^]^ or “structural disorder”^[^ ^85^ ^]^ in those studies but defined as “step size” in this work) sequentially, to the positions of particles in a perfect lattice, thereby creating a “disordered” distribution (i.e., disordering the lattice), while preserving the total number of particles in the system. While this method allows for the generation of increasingly larger disturbances from a perfect lattice and obtains a broad spectrum of distributions with increasing the allowed displacements, δ, it falls short in quantifying the disorder of the overall distribution. It also leads to what is misconstrued as a disorder parameter, δ, and the resulting generated distributions depend on the starting lattice (HPC, FCC, BCC, etc.) thus lacking universality due to the loss of a fixed reference. In other words, the distribution generated with the same value of δ can have different values for *T* and different values for *Q* order parameters using different lattices. In contrast, in the proposed method, T and Q provide a universal definition for any given disorder arrangement (e.g., biological or engineered as depicted for some example cases pertaining to this study in Figure 5). In broad terms, we can state that the perturbation method relies on displacement to regulate disturbance which cannot be used to quantify the arrangement of the architecture (e.g., tube, particles, or other features such as size, shape, and orientation).

Another commonly used method that has been used to generate and characterize disorder in literature is the Voronoi tessellation method (Figure 6b). This method involves random sequential additions of seed points within a domain, ensuring that each seed point maintains a minimum distance, *d_min_*, from one another. The space is then partitioned into Voronoi cells based on proximity to these seed points, resulting in a network of irregularly shaped cells that reflect the randomness of the seed placement. In the literature, the regularity parameter which is defined as α  =  *d_min_*/*d_HCP_* has been commonly used as a ‘disorder parameter’ where the value of 0 and 1 corresponds to the random and perfect lattice (HCP in 2D), respectively. Hence, these studies have generated disordered architected designs by considering the regularity parameter as a disorder parameter and varying it between 0 and 1. However, when we evaluate the translational and orientational order parameters for the designs with different regularity parameters, α (as shown in Figure 6c,d), we observed that the regularity parameter does not cover the entire spectrum of disordered distributions, and indeed remains in a narrow band closer to random. The *T* value remains nearly constant and nearly zero for the α value varying from 0 to 0.8, which shows all the distributions generated with different α values are nearly random. In other words, increasing α from 0 to 0.8 does not increase the translational order of the distribution. This is an interesting validation that the Voronoi tessellation method can only generate arrangements that are essentially almost always random from a translational order parameter, *T*, perspective. Yet, this limitation of the method should not be surprising since this algorithm works by “random Poisson's distribution”.^[^ ^92^ ^]^ This indicates that what is currently being considered as “order parameter, α” is indeed completely random and is only capable of generating arrangements that from a translational order parameter perspective are statistical noise and quite narrow in distributions (Figure 6e). This high‐level finding alludes to the inappropriate marking of α as order parameter for which a “perturbation parameter” nomenclature is more fit. Similarly, the *Q* value remained close to 0.42 (*Q* value for random distribution in 2D) for the α value varying from 0 to 0.6 providing a slightly larger range from an orientational order parameter point of view, Q, but nevertheless, limited even in the range to only highly disordered side of the spectrum (Figure 6e). The radial distribution function, *g*_2_(*r*) of the arrangements produced with the Voronoi Tessellation method represents values close to 1 which further indicates a random distribution, further reinforcing the findings based on the *T* and *Q*. (Figure S4i–p, Supporting Information).

It should be further noted that the Voronoi tessellation method is also less efficient in the event of generating the arrangement, for higher values of α (α > 0.8) in terms of computational time as it becomes increasingly intensive to generate and maintain the minimum distance constraint (*d_min_*) between seed points. This is due to the fact that as α increases, ensuring that each new seed point added does not violate the minimum distance requirement requires more checks and iterations, significantly increasing the computational time required to complete the distribution.

Considering the computational limitation of the method and the relation of α with *T* and *Q*, we can conclude that the Voronoi tessellation method can be used to only generate almost random or highly disordered distributions (Figure 6e) and not quantify disorder. More specifically, we have shown that the parameter α widely understood and taken as the “disorder parameter” not only does not quantity disorder but also is not able to generate arrangements that encompass the entire spectrum of disordered distributions or designs.

Therefore, the classical categorization of periodic versus non‐periodic or current algorithms for generating disorder (based on the regularity parameter α, or perturbation parameter δ) in studying or developing architected materials^[^ ^83^, ^94^, ^95^ ^]^ suffer from an understanding and quantification of disorder in the first place that prevents appropriate probing of the disorder‐property relationship. Additional shortcomings including the inability to capture and even generate a broad range of disorder spectrums and the role such spectrum plays in the design of otherwise unexplored domains can hinder the field from advancements in understanding new properties and discovering new interactions of materials arrangement with other fields (stress intensity, damage, crack, wave, etc.).

## 4 Conclusion

In conclusion, conventional cement‐based materials exhibit brittle failure under fracture due to limited toughening mechanisms such as uncracked ligament bridging^[^ ^96^ ^]^ and microcracking.^[^ ^97^ ^]^ As such microstructure of concrete suffers from limited energy absorption capacity and low resistance to cracking (low fracture toughness). This has limited the reliance on tensile and flexural properties of cement‐based materials, has imposed design constraints (reliance on and designing for compressive properties), and has imposed limits on the design landscape. Here, we propose engineering intentional tubular defects into cement‐based materials to tailor the properties of monolithic counterparts to enhance the specific fracture toughness. The underlying competition between tube size and shape enables the engineering of stepwise cracking, which in turn significantly enhances fracture toughness through rising R‐curves.

The findings of this study can, in principle, be extended to quasi‐brittle mortar and concrete, contributing to the formulation of a strategy for enhancing the fracture toughness of concrete materials through deliberate design and harnessing defects, enabling the creation of crack‐resistant characteristics in brittle and quasi‐brittle cement‐based materials. Deployment of these types of designs across size scales can be achieved, for instance, through leveraging the growing additive manufacturing techniques with cement paste at the desktop level to mortar and concrete at the robotic fabrication level.^[^ ^98^, ^99^, ^100^, ^101^, ^102^, ^103^, ^104^ ^]^ The use of statistical mechanics can help quantify the spectrum of disorder in the material, thus enabling a higher design level, as a means to engineer certain interaction characteristics with the material.

A statistical mechanics approach to quantifying disorder in architected materials can inform a novel approach to the design of not only the material but also the degree of disorder it contains. It can promote the qualitative assessment of the design‐performance relationship as it can elegantly help capture the orientation and translational aspects of geometry. Thus, this approach can be insightful for understanding the mechanics‐geometry relationships across various classes of architected materials (from brittle to ductile) and meta‐materials that interact with a field (stress, wave, damage, etc.), intuitively or exhaustively. The utility of this approach and proposed algorithm can be further unleashed by broadly or specifically applying it to understanding geometry in the first place or using it to design and achieve certain disorders to reverse engineer the mechanics of interest. Using a quantifiable and mathematically conceivable representation of the arrangement with the proposed disorder parameters can, for instance, more specifically answer questions with regard to optimality for strong, tough, or damage‐resilient designs in extreme or ultimate states in a more refined fashion in the context of the arrangement of material(s) or phase(s). These questions regarding the mechanics of material have generally been asked in the context of periodicity and non‐periodicity and existing methods to generate disturbance. Nevertheless, they may be revisited from a statistical mechanics approach to disorder for design purposes to discover new mechanisms, principles, and insights beyond bio‐inspired schemes.

## 5 Experimental Section

### Preparation of Cement Paste

Cement paste is composed of commercially available Type I cement acquired from Buzzi Unicem (Stockertown, PA, USA), deionized water, high range water reducing admixture (HRWRA, MasterGlenium 7700), and viscosity modifying admixtures (VMA, MasterMatrix 362). The cement paste is prepared by mixing 250 g cement, 71.5 g water, 1.84 g HRWRA, and 2.38 g VMA using the Twister Evolution Venturi vacuum mixer (Renfert). The mixing is performed using a two‐step mixing procedure.^[^ ^98^, ^99^ ^]^ The first step involves pre‐mixing for 25 s, followed by mixing at 400 rpm for 90 s at 70% vacuum. The second step of mixing was conducted at 400 rpm for 90 s at 100% vacuum.

### Hybrid Manufacturing

The tubular specimens are fabricated employing hybrid 3D‐printing and casting techniques (Figure 2). The first step involves 3D‐printing of positive mold using Polyvinyl alcohol (PVA) with the Ultimaker S5 as shown in Figure 2a. Subsequently, two‐component urethane rubber (VytaFlex 60) was mixed in 1:1 by weight. The mix is then poured into the PVA mold to form the negative mold of the tubular specimen. Immediately following the pouring, the PVA mold filled with liquid urethane rubber is placed into a vacuum chamber for 10 minutes to extract the air bubbles from the liquid urethane rubber. The urethane rubber is then left to harden for at least 14 h at 35 ± 5% relative humidity and 25 ± 3 °C. The urethane rubber mold is then obtained by keeping the molds in the ultrasonic bath at 90° C for 12 hours to dissolve the PVA (Figure 2b). The fresh cement paste is then poured into the urethane rubber mold to cast tubular architected materials (Figure 2c). The specimens are fabricated in a laboratory environment at 21 ± 3 °C and relative humidity of 45 ± 5%. Then, they are transferred to a curing chamber maintained at a relative humidity of 97 ± 2% using saturated solutions of potassium sulfate,^[^ ^105^, ^106^ ^]^ where they undergo a 7‐day curing process.

### Mechanical Properties Characterization

The modulus of rupture (MOR) is characterized using the three‐point bending test (3PB) according to ASTM C293M‐16.^[^ ^107^ ^]^ The fracture toughness is determined using the single‐edge notch bend (SENB) according to the following ASTM E1820‐20b.^[^ ^108^ ^]^ The unnotched and notched prismatic specimens of 40 × 40 × 130 mm dimensions are prepared for 3PB and SENB testing, respectively. In SENB specimens, a 2 mm deep notch was first introduced using a 2 mm thick diamond saw followed by an additional 2 mm depth using the razor of 0.3 mm thickness. The loading rates of 0.1 and 0.05 mm min^−1^ are employed for 3PB and SENB testing, respectively. Both mechanical tests are performed using the electromechanical frame (MTS Criterion C45.305) with a load cell of 20 kN capacity. At least three repetitions of each type of specimen are performed and the average value is reported. The details of the calculations involved for each test are discussed in Supporting Information.

### Statistical Analysis

Data is presented as mean ± SD. A minimum of 3 repetitions were performed for each test. The one‐tailed F‐test and T‐test with a confidence interval of 95%, were employed as a statistical method to study the significant difference between variance and mean of the data, respectively. *p* < 0.05 indicates the statistically significant difference between the samples. α value of 0.05 was used. Data Analysis Toolbox in Microsoft Excel was used to perform statistical analysis.

## Conflict of Interest

The authors declare no conflict of interest.

## Author Contributions

S.G. and R.M. conceived the project. S.G. conducted the experiments and analysis of data. S.G. and R.M. interpreted the data. S.G. and R.M. wrote and edited the original manuscript, and R.M. acquired funding.

## Supporting information



Supporting Information

Supplemental Movie 1

Supplemental Movie 2

## Data Availability

The data that support the findings of this study are available from the corresponding author upon reasonable request.
