# Supplementary material for: Tough Cortical Bone‐Inspired Tubular Architected Cement‐Based Material with Disorder
Source: Adv Mater. 2024 Sep 10;36(52):2313904. doi: 10.1002/adma.202313904 (PMC11681317; doi:10.1002/adma.202313904)
Supplement: Supplementary file 1 — Supporting Information [file ADMA-36-2313904-s001.docx]

Supporting Information

**Tough Cortical Bone-Inspired Tubular Architected Cement-based Materials**

*Shashank Gupta, Reza Moini**

Shashank Gupta, Reza Moini

Department of Civil and Environmental Engineering, Princeton University, Princeton, NJ, USA

Email: [Reza.Moini@princeton.edu](mailto:Reza.Moini@princeton.edu)

**Characterization of Fracture Toughness and Modulus of Rupture**

*Fracture Toughness*: The fracture toughness is determined using the single-edge notch bend (SENB) according to the ASTM E1820-20b.^[1–10]^ The crack-initiation fracture toughness, $K_{Ic}$, is defined by Eq. S1.

$$K_{Ic}=\frac{P_{c}L}{WD^{1.5}}*f\left( \frac{a_{o}}{D} \right) (Eq. S1)$$

where, $P_{c}$ is the load corresponding to the initiation of the crack. *L*, *W*, *D,* and $a_{o}$ denote the span length, width, depth, and notch length of the prismatic beam, respectively. The $L$ , *W*, and *D* of the beams are 120 mm, 40 mm, and 40 mm, respectively. The notch length-to-depth ratio $(\frac{a_{o}}{D})$ of 0.1 is employed.^[11]^ The function $f\left( \frac{a_{o}}{D} \right)$ considers the geometrical parameter (shape function) of the SENB specimen and the notch and is defined by Eq. S2. The *L/D* ratio of 3.0 was used here which was considered for adjusting the value of shape function.^[5,7,8,12,13]^

$$f\left( \frac{a_{o}}{D} \right)=\frac{3\left( \frac{a_{o}}{D} \right)^{\frac{1}{2}}\left( 1.99-\left( \frac{a_{o}}{D} \right)\left( 1-\frac{a_{o}}{D} \right)\left[ 2.15-3.93\left( \frac{a_{o}}{D} \right)+2.7\left( \frac{a_{o}}{D} \right)^{2} \right] \right)}{2\left( 1+\frac{2a_{o}}{D} \right)\left( 1-\frac{a_{o}}{D} \right)^{\frac{3}{2}}} (Eq. S2)$$

The *J*-integral determines the strain energy release rate for the crack in the material. The computation of the *J*-integral involves two components: the elastic component ($J_{el}$) and the plastic component ($J_{pl}$) The $J_{el}$ and $J_{pl}$ are defined by Eq. S3 & S4, respectively.

$$J_{el}=\frac{K_{Ic}^{2}}{E'} (Eq. S3)$$

where, $K_{Ic}$ is the crack initiation fracture toughness. $E'$ is the plane strain Young’s Modulus of the hardened cement paste (30 GPa).^[14–16]^

$$J_{pl}=\frac{\eta_{pl}A_{pl}}{W(D-a_{0})} (Eq. S4)$$

where $\eta_{pl}$ is the dimensionless function of geometry and is defined as 1.9 when load-line displacement is used for SENB specimen as per ASTM E1820-20b, $(D-a_{0})$denotes the remaining ligament length, and $A_{pl}$ is the area under the force-displacement curve beyond the load corresponding to the initiation of the crack ($P_{c})$ as shown by Eq. S5.

$$A_{pl}=\sum_{i=P_{c}}^{n} P_{i}\times\delta_{i} (Eq. S5)$$

The fracture toughness, $K_{J}$, can be further defined as shown in Eq. S6.

$$K_{J}=\sqrt{{(J}_{el}+J_{pl})E'} (Eq. S6)$$

The $K_{J}$ can be plotted for the incremental points of crack extension, $a_{i}$, which is defined by Eq. S7.

$$a_{i}=a_{i-1}+\frac{D-a_{i}}{2} \frac{C_{i}-C_{i-1}}{C_{i}} (Eq.S7)$$

where, $C_{i}$ is the instantaneous compliance of the notched specimen and it can be defined by Eq. S8 as the function of the ratio of crosshead displacement ($u_{i}$) to applied load ($P_{i}$).

$$C_{i}=\frac{u_{i}}{P_{i}} (Eq. S8)$$

The extension of the crack tip corresponds to an increase of $A_{pl}$ which allows plotting of the growth of fracture toughness against crack extension $a_{n}$ as a resistance curve (R-curve). The representation of fracture toughness in relation to the crack extension (R-curve) plot is valuable in providing an understanding of the evolution of the material's resistance to crack initiation and propagation.

According to ASTM E1820-20b ^[1]^, the maximum J-integral capacity is defined by Eq. S9.

$$J_{max}=min\left\{ \frac{W\sigma_{y}}{10},\frac{(D-a_{0})\sigma_{y}}{10} \right\} (Eq. S9)$$

On the other hand, the maximum crack extension for the specimen is defined by Eq. S10 according to ASTM E1820-20b.^[1]^

$$a_{max}=0.25\left( D-a_{o} \right) (Eq. S10)$$

The $J$ value corresponding to the maximum crack extension ($a_{max}$) is lower than the $J_{max}$ (obtained from Eq. S9) for all the monolithic and tubular architected specimens in this study. The $J$ value corresponds to $a_{max}$ is used to determine the ASTM limit of fracture toughness of the material.

*Modulus of Rupture*: The modulus of rupture, MOR, is determined using the three-point bending test (3PB) of the unnotched prismatic specimen according to ASTM C293M-16.^[17]^ The maximum peak load, $P_{max}$, is used to calculate the MOR as shown by Eq. S11.

$$MOR= \frac{3\left( P_{max} \right)L}{2WD^{2}} (Eq. S11)$$

where, $L$ , *W*, and *D* denote the span length, width, and depth of the prismatic beam, respectively. The $L$ , *W*, and *D* of the beams are 120 mm, 40 mm, and 40 mm, respectively.

**Characterizing the *Degree of Disorder* in Architected Materials Using Statistical Mechanics**

Tubular architected material can be designed with various arrangements. In this study, the spatial distribution of the tubes is characterized and quantified using the radial distribution function and two-order parameters. The radial distribution function ($g_{2}(r)$) is the probability distribution of locating the point at a radial distance ($r$) from the center of a reference point similar to those applied in the molecular structure of gas and liquids.^[18]^ Furthermore, the degree of disorder is quantified by two order parameters (translational and orientational) both of which are scalar quantities that represent the value of zero for a completely random distribution (e.g., particle distribution in an ideal gas) and 1 for a perfectly ordered distribution (e.g., crystal lattice).^[18–21]^ Translational and orientational order parameters have been widely used to study the spatial distribution of molecules in liquid crystal and amorphous solids,^[19,20]^ but have rarely been adopted to rigorously characterize a materials architecture beyond the qualitative periodic/non-periodic metrics.

The translational order parameter (*T*) measures the spatial ordering of particles relative to the perfect Hexagonal closed packing (HCP) lattice and the ideal gas, at the same particle density, and is defined by Eq. S12.^[18]^

$$T= \left| \frac{\sum_{i=1}^{N_{s}} \left( n_{i}-{n_{i}}^{ideal} \right)}{\sum_{i=1}^{N_{s}} \left( {n_{i}}^{HCP}-{n_{i}}^{ideal} \right)} \right| ( Eq.S12)$$

where, $n_{i}$ denotes the average occupation number for the shell of thickness $\Delta d_{HCP}$ which is centered at the distance from a reference sphere that equals the distance of the $i$th nearest neighbor for the HCP lattice of the same particle density; $d_{HCP}$is the first nearest-neighbor distance for the HCP lattice; $N_{s}$ is the total number of shells; and ${n_{i}}^{HCP}$ and ${n_{i}}^{ideal}$ are the occupation numbers of the HCP lattice and ideal gas, respectively.

**Figure S1**. a-c) Random, disordered, and ordered distribution of particles, respectively, and the corresponding d-f) schematic of radial distribution functions, and g) the range of translational and orientational order parameters.

On the other hand, the orientational order parameter is defined by spherical harmonics ($Y_{lm}$), which are used in statistical mechanics to describe the angular distribution of particles. The orientational bond parameter, $Q_{6}$, (where, $l=6$) is defined by Eq. S13.^[20,21]^

$$Q_{6}=\left( \frac{4\pi}{13}\sum_{m= -6}^{6} \left| \bar{Y}_{6m} \right|^{2} \right)^{1/2} ( Eq. S13)$$

The orientational order parameter, $Q$, is defined as the orientational bond order parameter, $Q_{6}$, normalized by its value for perfect hexagonal close packing (HCP) lattice, $Q_{6}^{HCP}$. The orientational order parameter, $Q$, is expressed by Eq. S14.

$$Q= \frac{Q_{6}}{Q_{6}^{HCP}} (Eq. S14)$$

The radial distribution function and the corresponding two-order parameters (*T*, $Q$) for random (ideal gas), disordered, and perfectly ordered (HCP) distribution are shown in **Figure S1**.

**Figure S2**. **Radial distribution function of circular and elliptical tubular designs**. a,b) Designs and corresponding c,d) normalized radial distribution function of circular and elliptical tubular architected materials.

The designs of circular and elliptical architected materials of different porosities ($\varphi$ = 20%, 30%, 40%, & 50%) and aspect ratios ($e$ = 2, 2.5, & 3.0) are presented in **Figure S2**a, b. The radial distribution functions, $g_{2}(r)$, are calculated for the (dissimilar) distribution of the center of tubes in the circular and elliptical architected materials. The normalized values of $g_{2}\left( r \right)$ with respect to peak value are presented for circular and elliptical architected materials in Figure S2c, d, respectively. In this study, the normalized $g_{2}(r)$ values for architected materials are benchmarked against those of the HCP lattice (Figure S2c, d). The findings reveal that, in terms of radial distance, the peak values of the normalized $g_{2}(r)$ for both circular and elliptical tubular materials do not align with the HCP lattice.

To capture the variation of the statistical parameter with degree of disorder, the translational (*T*) and orientational (*Q*) order parameters were determined for the range of 2D distributions of the particles, ranging from perfectly ordered, to disordered, and to random. In order to generate a broad range of disordered distribution of particles bound by random and perfectly ordered limits, the Metropolis algorithm for hard particles was employed.^[18]^ In this algorithm, firstly, an initial configuration of the N particles is generated in a 2D bounding box such that no particles overlap one other. Then, a new configuration of particles is generated by moving a particle along each axis (x and y-axis) by a displacement, randomly distributed in the interval of $[-\delta, \delta]$, where $\delta$ is the maximum allowable ‘step size’ (the limit of the displacement). In the new configuration, if the displaced particle does not overlap with another particle, the move of the particle is accepted, otherwise, it is rejected. This process of moving the particle and accepting/rejecting the move is then repeated for all the N particles in the box to conclude one iteration. In our study, the initial configuration of the HCP lattice (perfectly ordered system) was chosen, and the degree of disorder was increased by increasing the *allowable* (maximum) step size ($\delta$) for displacing the particle.

As a result, choosing an HCP lattice for the initial configuration allows generating the particle distribution varying from ordered to disordered by increasing $\delta$ (**Figure S4a-d**). It should be further noted that the periodic boundary conditions were used to calculate the statistical parameters ($T$, $Q$, and $g_{2}(r)$), to use the small unit cell to approximate the large system of particles.


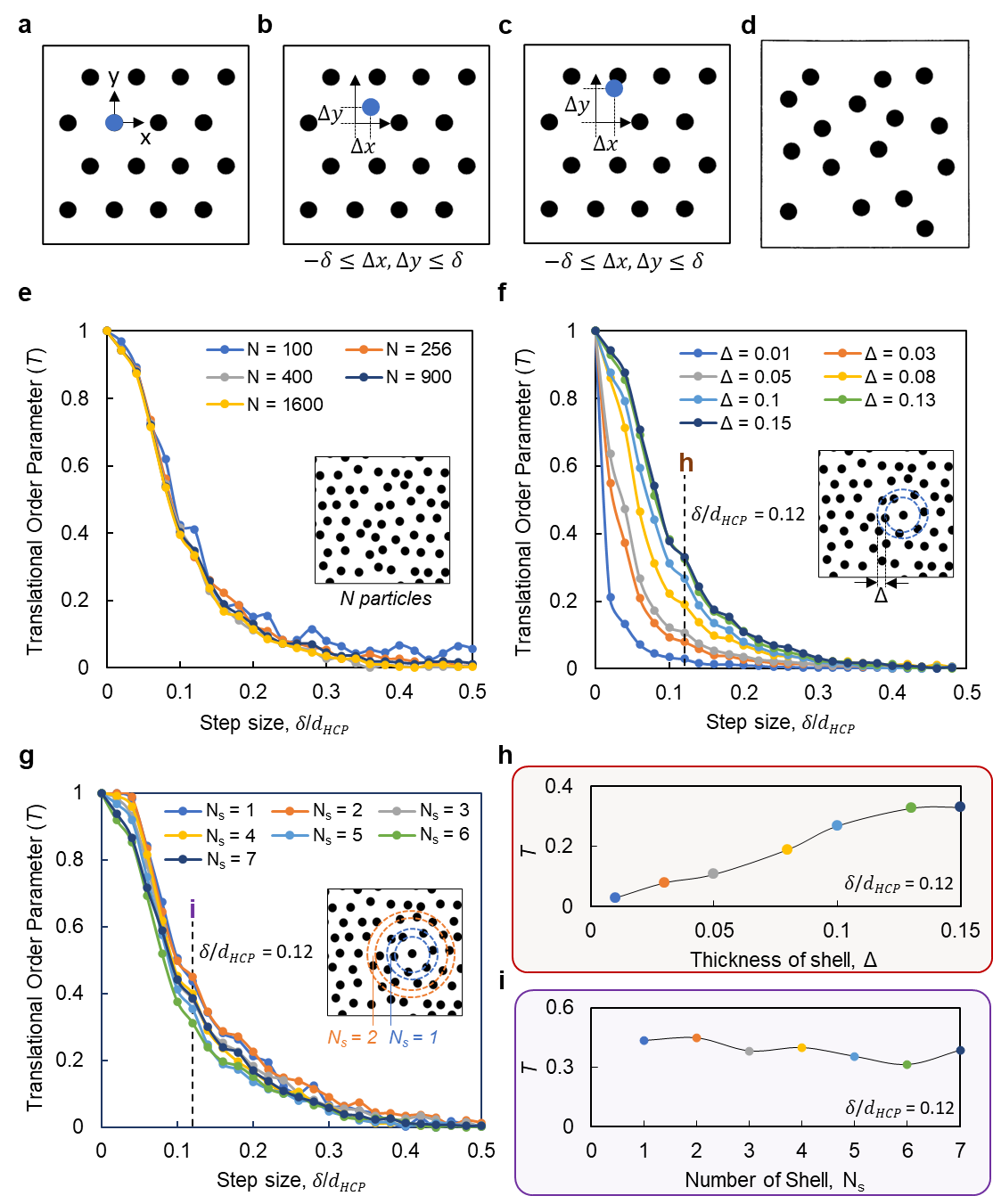


**Figure S3**. **Schematics of the Metropolis algorithm generates a disordered distribution** from a) an initial hexagonal close packing (HCP) lattice by moving a particle with an allowable step size $\delta$. A particle displacement is b) accepted if there is no overlap with another particle and c) rejected otherwise. d) The process repeats for all particles (one iteration) to generate a disordered distribution bound by HCP distribution and 2D random distribution. Parametric study of the translational order parameter (*T*) as a function of normalized step size, $\delta/d_{HCP}$ for varied e) number of particles or points, N, (f, h) thickness of the shell, $\Delta$, and (g, i) number of shells $N_{s}$.

The translational order parameter ($T$) depends on three variables, the number of particles in the unit cell ($N$), the thickness of the shell ($\Delta$), and the number of shells ($N_{s}$).^[20,21]^ Therefore, to calculate the $T$ parameter for particle systems with different degrees of disorder, a parametric study was performed with three variables ($N, \Delta$, & $N_{s}$) as exhibited in **Figure S3**. It should be noted that $Tthe$ parameter is 1 for the HCP lattice at $\delta=0$. As $\delta$ approaches $0.5d_{HCP}$ for all the different values of parameters, *T* approaches 0, as shown in Figure S3a-c.

The increase in the number of particles in the unit cell ($N$) reduces the statistical noise in the $T$ parameter, as shown in Figure S3a, but rapidly increases the computational time. Therefore, considering the balance between the low statistical noise and the computational time, the number of particles in a unit cell was comfortably chosen as 900.

The shell size thickness, $\Delta$, controls the occupation number, $n_{i}$, in a single shell used for the calculation of the *T* parameter, as shown in Figure S1a-c. Increasing the $\Delta$ value higher than 0.15 can merge two (or more) surrounding peaks of $g_{2}(r)$ of the HCP lattice into one peak, meaning that the particles at two different distances in the HCP lattice will lie in the single shell. Therefore, 0.15 was kept as the upper limit of $\Delta$. It can be noticed from Figure S3b, that the $T$ parameter increases with increasing $\Delta$. Furthermore, at the fixed value of $\delta$, say $\delta=0.12d_{HCP}$, the $T$ parameter increases with $\delta$ at the decreasing rate and converges to 0.33 at the $\Delta=0.15$ as shown in Figure S3d. Considering the convergence, $\Delta=0.15$ was selected for the calculation of $T$ parameter. In the context of the number of shells, $N_{s}$, the $T$ parameter did not show any trend with the increasing $N_{s}$ as exhibited by Figure S3c. At the fixed value of $\delta$, say $\delta=0.12d_{HCP}$, it was found that $T$ parameter remained nearly constant. In this study, we have selected $N_{s}=7$, based on the literature.^[20]^


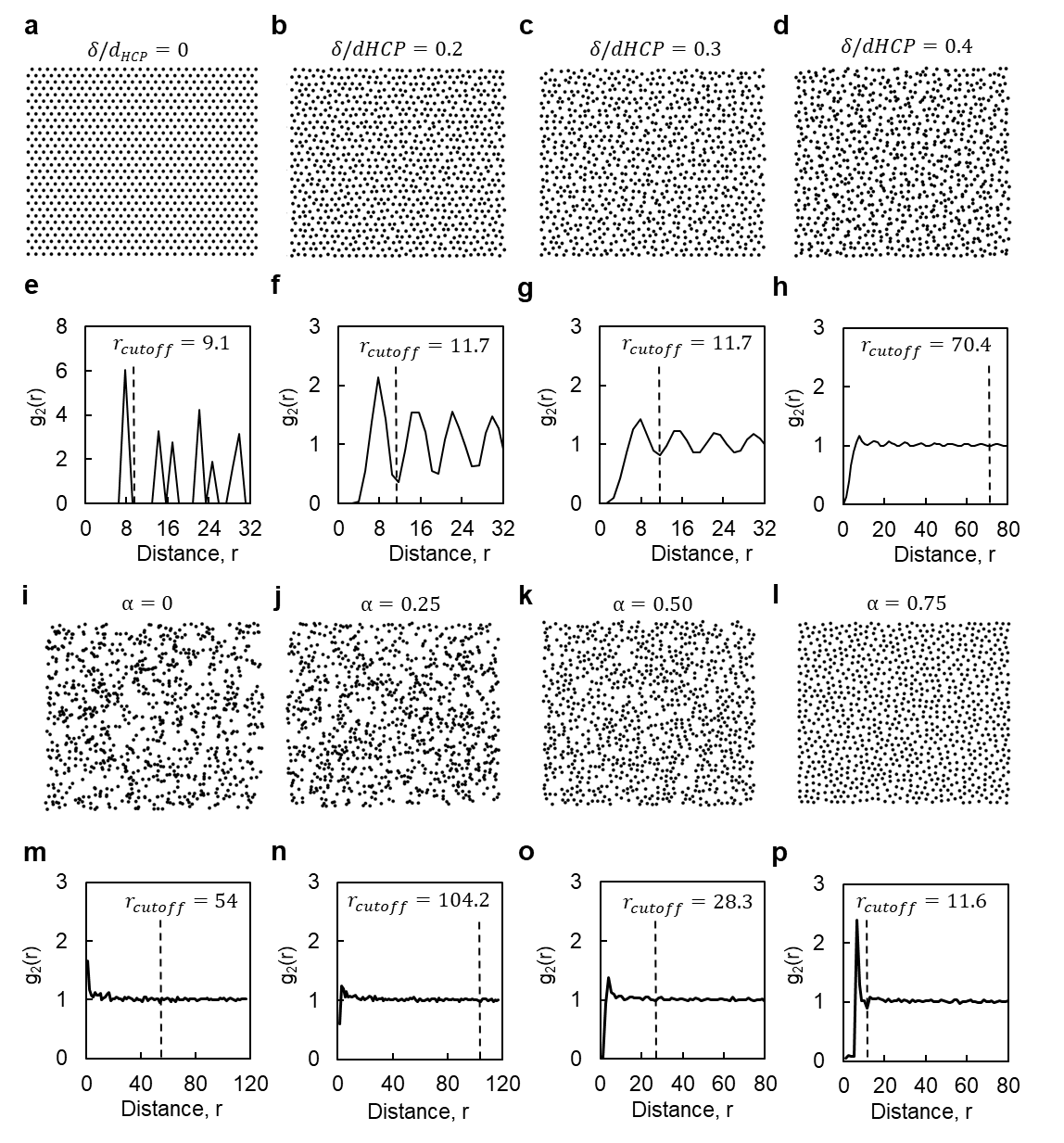


**Figure S4**. **Radial distribution functions for distributions generated from perturbation and Voronoi tessellation methods.** a-d) 2D – distribution of the 900 particles in a unit cell for maximum step size, $\delta$ varying from 0 to $0.4d_{HCP}$, e-h) the corresponding radial distribution function, $g_{2}(r)$ along with the cutoff radius, $r_{cutoff}$ (first minimum following the first peak in $g_{2}(r)$, i-l) 2D – distribution of the 900 particles for regularity parameter, $\alpha$ varying from 0 to $0.75$, and m-p) the corresponding radial distribution function, $g_{2}(r)$ along with the cutoff radius, $r_{cutoff}$ (first minimum following the first peak in $g_{2}(r)$.

The orientational bond parameter ($Q_{6}$) depends on the total number of neighboring particles lying within the cutoff radial distance, $r_{cutoff}$, which is considered the first minimum following the first peak in $g_{2}(r)$.^[20,21]^ As the degree of disorder in the system increases (with increasing $\delta/d_{HCP}$), the $r_{cutoff}$ increases, as shown by Figure S4e-h, It is worth noting that the $r_{cutoff}$ value increases by 7.7 times as the $\delta$increases from 0 for the HCP lattice to $0.4d_{HCP}$ for a highly disordered system.

Considering the determined values from the parametric study, *T* and *Q_6_* are determined for the tubular architected materials. Both the circular and elliptical architected material exhibit low translational order parameters$, T,$0.21 and 0.30, respectively, which suggest that the periodic distribution of architecture has similar translational order parameters as particles in amorphous glasses.^[20]^

On the other hand, the circular architected materials exhibit an orientational order parameter$, Q_{6}$ = 0.77, which is higher than the $Q_{6}$ = 0.44 quantified for elliptical architected materials. The $Q$ of circular and elliptical architected materials are closer to the distribution of particles in crystal and amorphous glass, respectively.^[20]^ The findings indicate for the first time, that for seemingly periodic design (distributions of features) in tubular architected materials, $Q$, more clearly represents the degree of order than $T$ for periodic distributions.

The use of the radial distribution function, along with the order parameters, alludes to the fact that although circular and elliptical tubular architecture appear periodic in terms of distribution, they are not perfectly ordered. This is counter-intuitive and indicates that periodicity is an insufficient measure to represent material distributions. Moreover, the spatial distribution of circular architected materials represents a higher orientational order parameter closer to a perfectly ordered arrangement (HCP) compared to the elliptical counterparts.

The findings can be examined and extended to other types of material distributions to understand the effectiveness of *Q* vs. *T* in capturing the degree of disorder in the field of architected materials. More importantly, the proper application of statistical mechanics and ‘degree of disorder’ can help quantify the degree of order and suggest a better measure than the qualitative assessment of ‘periodicity’. In other words, the period vs. non-periodic conceptualization falls short of capturing the spectrum of degree of ‘order’ in the material (e.g., periodic distributions can be disordered whereas non-periodic distribution can have a very wide range of degrees of disorder which can determine the mechanical and wave propagation properties, none of which are captured by periodicity). In addition, the periodic vs. non-periodic classification is binary and lacks the capture of the spectrum that can be present in material arrangements.

The disordered designs in tubular geometries, orientations, and distributions can have the potential impact on material strength and fracture toughness. Our future work will focus on exploring a broader design domain encompassing variations in porosity, tube shape, and orientation, alongside a range of practical, manufacturable distributions. We aim to incorporate disorder parameters, *T* and *Q*, as the key factors in characterizing these designs. Additionally, we plan to enhance our theoretical framework with crack propagation studies supported by numerical simulations, paving the way for innovative approaches to designing non-periodic and disordered systems in brittle materials. This initial study with the periodic distribution of the tubes serves as a foundational step towards employing circular and elliptical voids to optimize fracture properties, setting the stage for further investigation into more complex, polydisperse systems.

**Mechanical Response of Circular Architected Materials**

The effect of porosity on the fracture response and MOR of tubular architected materials with circular design is presented in **Figure S5**. The load-displacement plots obtained from the single-edge notch bend (SENB) test reveal distinct mechanical responses among various specimens (Figure S5a). The solid monolithic material, Circular, 20%, and Circular, 30% exhibit brittle failure. In contrast, Circular, 40% and Circular, 50% display stepwise cracking behavior characterized by multiple instances of hardening and softening. Notably, the crack-initiation load decreases with increasing porosity, *φ*, in tubular architected materials.

The R-curve, representing crack growth resistance ($\Delta$a) in terms of fracture toughness (K_J_), also shows significant differences in fracture response among specimens (Figure S5b). The solid material, Circular, 20%, and Circular, 30% display nearly flat R-curves, indicating limited crack growth resistance. Conversely, Circular, 40% and Circular, 50% exhibit stepwise increases in the R-curve, signifying multiple steep rises in fracture toughness followed by sudden crack extensions. Circular, 40% demonstrates the highest growth in K_J_, surpassing other designs and the solid counterpart.

A decreasing trend in average crack initiation fracture toughness (K_IC_) is observed with increasing porosity, aligned with the reduced crack initiation load in the load-displacement plots. Circular, 20% and Circular, 30% exhibit K_IC_ of $7.90 \pm1.10 MPa.{mm}^{0.5}$ and $6.84 \pm1.08 MPa.{mm}^{0.5}$, respectively, which are statistically similar to the solid material ($8.37 \pm1.36 MPa.{mm}^{0.5}$). However, Circular, 40% and Circular, 50% display K_IC_ of $4.04 \pm1.35 MPa.{mm}^{0.5}$ and $3.22 \pm0.85 MPa.{mm}^{0.5}$ which are significantly lower than solid (Figure S5c). Nevertheless, no significant difference between all circular tubular material vs. solid material is found when specific values (normalized with respect to density) of K_IC_ are used.

Intriguingly, despite decreasing K_IC_ values with porosity, Circular, 40% and Circular, 50% demonstrate a substantial increase in average crack propagation fracture toughness (K_JC_). The K_JC_ of Circular, 40% and Circular, 50% are $18.89 \pm6.02 MPa.{mm}^{0.5}$ and $11.27 \pm1.11 MPa.{mm}^{0.5}$which are significantly higher than the solid material by 2.25 and 1.34 folds. Circular, 20% and Circular, 30% demonstrate the K_JC_ of $7.90 \pm1.09 MPa.{mm}^{0.5}$ and $9.74 \pm1.85 MPa.{mm}^{0.5}$ which are statistically similar to solid (Figure S5d).

The Circular, 20%, and Circular, 40%, exhibit modulus of rupture (MOR) of $3.16 \pm0.36 MPa$ and $3.41 \pm0.68 MPa$ which are statistically similar to that of the solid counterpart ($3.71 \pm0.38 MPa$). However, Circular, 30% and Circular, 50% demonstrate MOR of $2.70 \pm0.22 MPa$ and $2.61 \pm0.50 MPa$ which are significantly lower than the solid material. The average MOR of tubular architected materials presents an expected decreasing trend with increasing porosity, except for Circular, 40% (Figure S5e). An F-test and a T-test with a confidence level of 95% are used for all statistical analyses.

The underlying response that generates the trend in fracture toughness is further elaborated in this Supporting Information (Theoretical and Experimental Analysis Section) by discussing the theoretical stress intensity factors (K_I_) in simplified analyses under far-field tension.


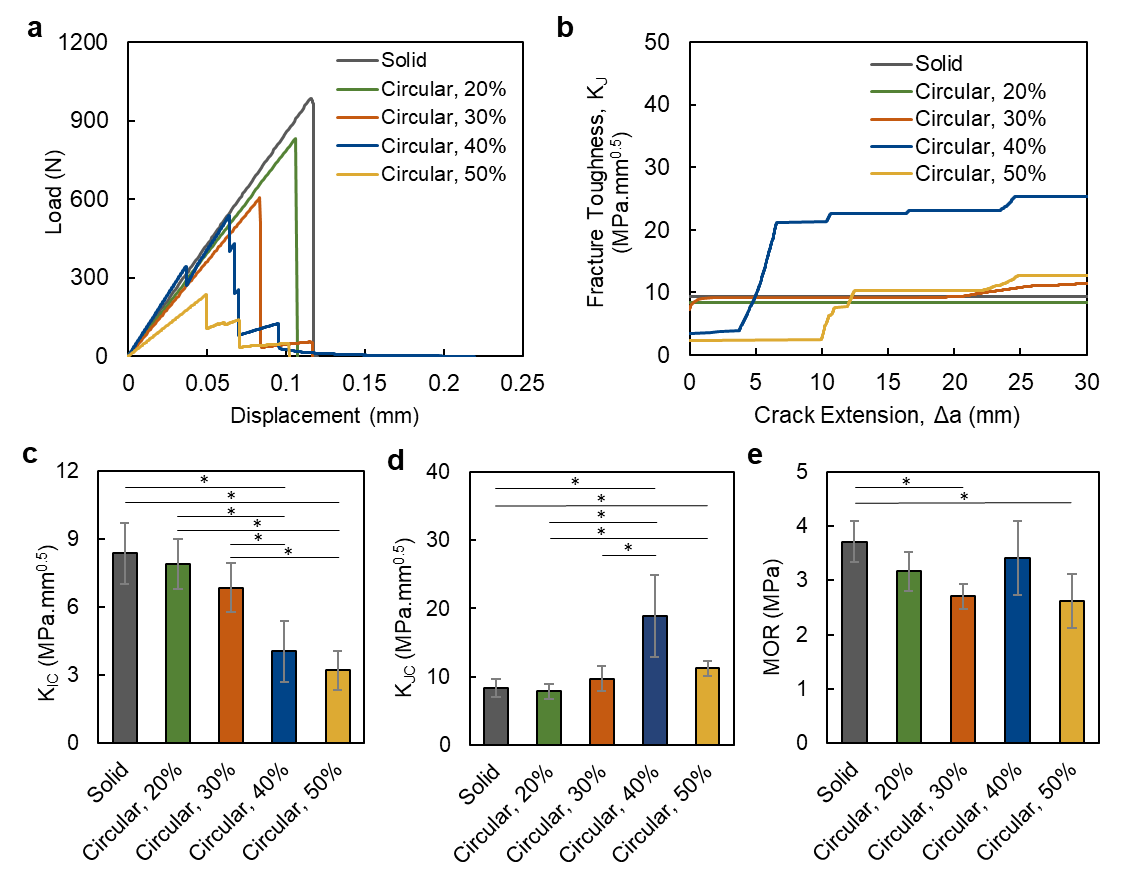


**Figure S5**. **Mechanical response of tubular architected cement-based material with circular design compared to monolithic solid.** a) Load – displacement plots obtained from SENB specimens, b) R-curve demonstrating the toughness vs. crack extension, c, d, and e) Crack initiation fracture toughness (K_IC_), fracture toughness (K_JC_), and modulus of rupture (MOR) of the architected material compared to monolithic solid. Data is shown as mean ± SD. ‘$*$’ depicts $p<0.05$ which indicates the statistically significant difference between the samples (at the ends of the solid line below a ‘$*$’). *p*-value is obtained from the F-test and T-test.

**Mechanical Response of Elliptical Architected Materials**

Figure S5 establishes that the tubular architected material with 40% porosity displays the highest fracture toughness compared to other circular designs. Hence, a porosity of 40% is employed to study the effect of the aspect ratio of tubes on the mechanical response of the tubular architected material. The aspect ratio, indicating the ratio of the semi-major axis to the semi-minor axis, is varied from 2 to 3 ($e$ = 2, 2.5, & 3) in this study.

The L-D plot in **Figure S6**a illustrates that solid material, Elliptical, 40% $e$ = 2, and Elliptical, 40% $e$ = 3 fail nearly in a brittle fashion whereas Elliptical, 40% $e$ = 2, $e$ = 2.5, $e$ = 3 demonstrate stepwise failure with $e$ = 2.5 outperforming other the cases. Consequently, Elliptical, 40% $e$ = 2.5 demonstrates a notable rise in the R-curve compared to other elliptical as well as the solid materials (Figure S6b).

**
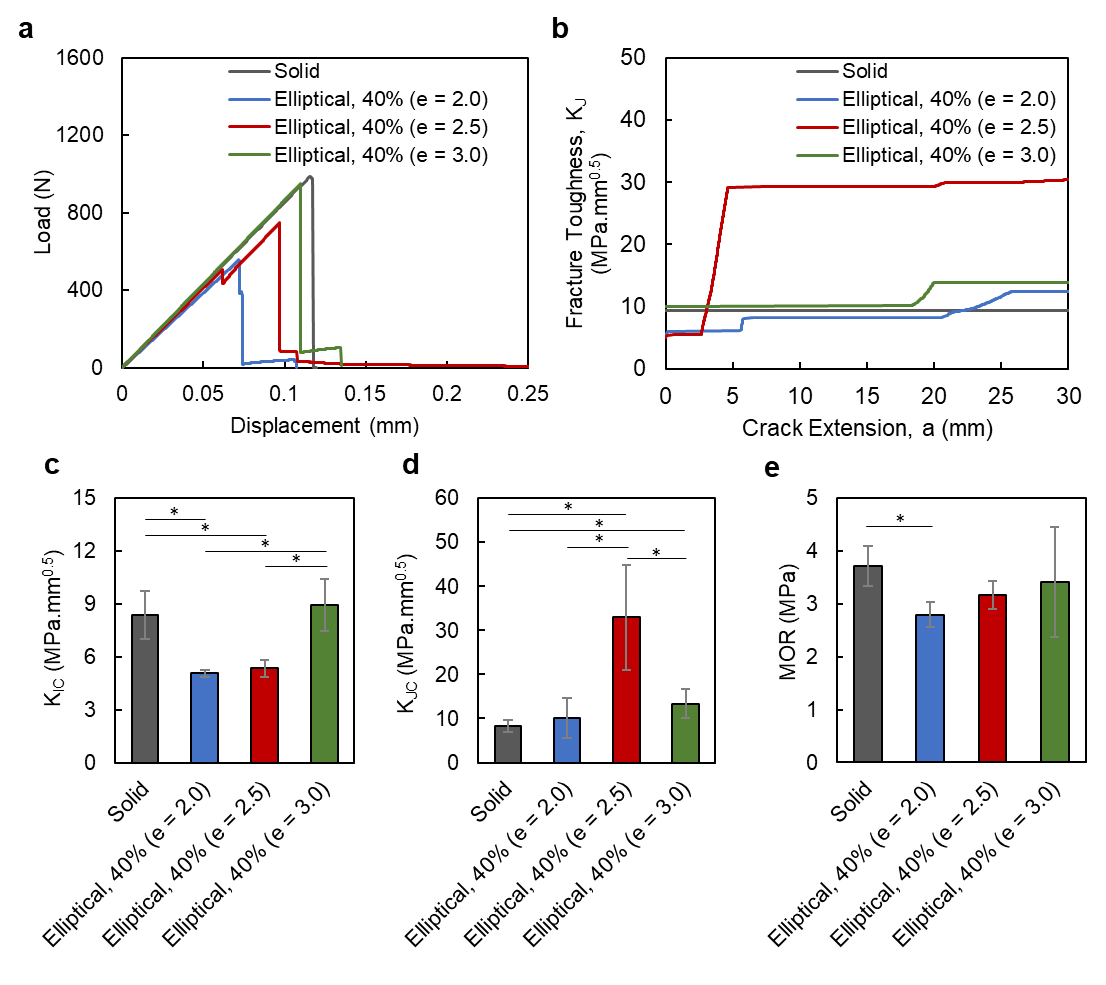
**

**Figure S6**. **Mechanical response of tubular architected cement-based material with elliptical design compared to monolithic conventionally cast solid.** a) Load – displacement plots obtained from SENB specimens, b) R-curve showing the toughness vs. crack extension, c, d, and e) Crack initiation fracture toughness (K_IC_), fracture toughness (K_JC_), and modulus of rupture (MOR) of the architected material compared to monolithic cast. Data is shown as mean ± SD. ‘$*$’ depicts $p<0.05$ which indicates the statistically significant difference between the samples (at the ends of the solid line below a ‘$*$’). *p*-value is obtained from the F-test and T-test.

Examining Figure S6c, we observe an increasing trend in average K_IC_ with aspect ratio,$e$, in tubular architected material. The K_IC_ of Elliptical, 40%, $e$ = 3 is $8.94 \pm1.47 MPa.{mm}^{0.5}$ which is statistically similar to the solid material. However, Elliptical, 40% $e$ = 2 and Elliptical, 40% $e$ = 2.5 exhibits K_IC_ of $5.05 \pm0.21 MPa.{mm}^{0.5}$ and $5.36 \pm0.48 MPa.{mm}^{0.5}$ which are statistically lower compared to solid material. On the other hand, the Elliptical, 40% $e$ = 2.5 demonstrates K_JC_ of $33.24 \pm11.90 MPa.{mm}^{0.5}$ which is significantly higher compared to solid and other elliptical designs. However, elliptical-designed tubular materials with *e* = 2.0 and *e* = 3.0 demonstrate K_JC_ of $10.09 \pm4.50 MPa.{mm}^{0.5}$ and $13.34 \pm3.28 MPa.{mm}^{0.5}$ compared to solid case (Figure S6d).

Additionally, the Elliptical, 40% $e$ = 2.5 and $e$ = 3 exhibit MOR of $3.17 \pm0.26 MPa$ and $3.41 \pm1.03 MPa$ which are statistically similar to solid, while the Elliptical, 40% $e$ = 2 displays MOR of $2.80 \pm0.23 MPa$ which is significantly lower compared to the solid material (Figure S6e). An F-test and a T-test with a confidence level of 95% are used for all statistical analyses.

**Figure S7**. a,c) R-curve representing the specific fracture toughness vs. crack extension (shows as a line) of Circular, 40% and Elliptical, 40% marked with b,d) crack extension ($\Delta a_{i}$) obtained from DIC analysis during fracture experiments (shown as circle points).

The crack extension, determined through load-line displacement and calculated using the compliance method, can be corroborated for further validation of the rising R-curve and accuracy of the crack extension, by comparing it with the crack extension obtained through Digital Image Correlation (DIC) analysis during fracture experiments. The crack extensions, $\Delta a_{i}$, denoted on the R-curve in **Figure S7**a and c, correspond to the stepwise crack progression from tube in one layer to the another in both Circular, 40% and Elliptical, 40%, from the DIC, as shown in Figure S7b and d, respectively. These $\Delta a_{i}$ are positioned in close proximity to the onset of each step of the crack growth in the X-axis of the R-curve, indicating that the calculated crack extension is well-aligned with the experimentally observed crack extensions from DIC analysis initiated at the tubes and extended into the subsequent layers.

**Figure S8.** Load vs. Crack mouth opening displacement (CMOD) plots of tubular architected materials with a) circular and b) elliptical designs. Circle marks indicate the crack initiation from the tube.

The stepwise crack progression in tubular architected materials is also shown in circular and elliptical designs using load vs. crack mouth opening displacement (CMOD) plots obtained from the SENB test in **Figure S8**a and b, respectively. The solid material and tubular architected material with a lower range of porosity (*φ* = 20% and 30%) demonstrate brittle failure. The elliptical designs with *e* = 2.0 and 3.0 exhibit single CMOD extension followed by a delayed brittle failure. On the other hand, the multiple extensions of CMOD associated with the extension of the crack along the depth are observed in Circular, 40%, Circular, 50%, and Elliptical, 40% *e* = 2.5. The crack propagation throughout the depth of the sample and increased CMOD takes place at the initiation of the crack from the tube as marked by circles.

The underlying mechanism for the effect of the geometric attributes of the tube (circular vs. elliptical) on the fracture toughness is further elaborated in this Supporting Information (Effect of the Shape and Location Section) by analyzing the theoretical stress intensity factor under far-field tension.

**Theoretical and Experimental Analysis of Stress Intensity Factor Due to the Presence of Tube Ahead of a Notch in Tubular Architected Materials**

The stress intensity factor characterizes the intensity of the stress field in a small region surrounding the crack tip.^[23]^ The stress intensity factor, $K_{I,Solid}$, of the crack tip in the monolithic elastic solid material under the far-field tensile stresses, $\sigma_{x\infty}$ (as shown in **Figure S9**a), is defined using the Eq. S15.^[24]^

$$K_{I,Solid}=\sigma_{x\infty}\sqrt{\frac{\pi c}{2}} (Eq. S15)$$

where c is the pre-existing crack (notch) length. The presence of tubes in the material alter the stress intensity factor, $K_{I,Tubular}$, of the notch tip .^[24,25]^ The effect of a single tube on the stress intensity factor of a crack tip (Figure S9b) is the first step to understand crack-tube interaction and is determined by using the stress-based approach in this study.^[24,25]^ The first order solution of stress intensity factor due to the presence of a single tube , $K_{I,Tubular}$, is determined using the Eq. S16.^[24]^

$$K_{I,Tubular}=\sqrt{\frac{2}{\pi c}}\int_{y_{1}}^{y_{2}} \sigma_{x} \left( x,y \right)\left( \frac{y-y_{2}}{y_{1}-y} \right)^{0.5}dy (Eq. S16)$$

where, $\sigma_{x} \left( x,y \right)$ is the stress on the pre-existing crack (notch) line in the uncracked body due to the hollow tube as illustrated in Figure S10b. $y_{1}$ and $y_{2}$ are the relative position of the closest and farthest crack tip with respect to the center of the tube, respectively, as shown in Figure S9b.

The $\sigma_{x} \left( x,y \right)$ is further determined under uniaxial far-field tension using the Eq. S17.^[26]^

$$\sigma_{x} \left( x,y \right)= \frac{\alpha+\bar{\alpha}}{2} (Eq. S17)$$

where,

$$\alpha= \frac{\sigma_{x\infty}}{4}\left[ \left\{ \left( A+\bar{A} \right)+(Bcoth\left( \zeta\right)+\bar{B}\coth\left( \bar{\zeta} \right)) \right\}-\frac{1}{{sinh}^{3}(\zeta)} \left\{ -\left( Bcosh\left( \bar{\zeta} \right)+Ccos\left( \zeta\right) \right)+4D{sinh}^{3}\left( \zeta\right)+2Ecosh(\zeta)(2{sinh}^{2}\left( \zeta\right)-1) \right\} \right] (Eq. S18)$$

and $\zeta$is the coordinate in the transformed plane and it is the function of semi-major and semi-minor lengths ($a$, $b$) of the elliptical tube with the center of tube at ($0$,$0$). $\zeta$ is defined by Eq. S19.^[26]^

$$\zeta=\cosh^{-1} \left[ \frac{x+iy}{\sqrt{a^{2}-b^{2}}} \right] (Eq. S19)$$

On the other hand, A, B, C, D, and E are the geometrical parameters determined by Eq. S20-S24.^[26]^

$$A =e^{2\xi_{0}} (Eq. S20)$$

$$B =1-e^{2\xi_{0}} (Eq. S21)$$

$$C =1-cosh(2\xi_{0}) (Eq. S22)$$

$$D =-\frac{1}{2}e^{2\xi_{0}}cosh(2\xi_{0}) (Eq. S23)$$

$$E =-\frac{1}{2}e^{2\xi_{0}}sinh(2\xi_{0}) (Eq. S24)$$

where $\xi_{0}$ represents the tube boundary and can be defined by Eq. S25.^[26]^

$$\xi_{0}=\cosh^{-1} \left[ \frac{a}{\sqrt{a^{2}-b^{2}}} \right] (Eq. S25)$$

The value of $K_{I,Tubular}$ obtained from Eq. S16 is validated by comparing them to values of $K_{I}$ provided by Tirosh and Tetelman, 1976,^[24]^ for circular tubes of different sizes used in this work.

Furthermore, the stress intensity factor ratio, $F\left( y_{1},y_{2} \right)$, can be defined as the ratio of $K_{I}$ of tubular architected material to that of solid and is defined by Eq. S26.

$$F\left( y_{1},y_{2} \right)=\frac{K_{I,Tubular}}{K_{I,Solid}}=\frac{1}{\sigma_{x\infty}}\frac{2}{\pi c} \int_{y_{1}}^{y_{2}} \sigma_{x} \left( x,y \right)\left( \frac{y-y_{2}}{y_{1}-y} \right)^{0.5}dy (Eq. S26)$$

The stress intensity factors, $K_{I}$, for solid, Circular, 40%, and Elliptical, 40% *e* = 2.5, as representative tubular cases, determined using Eq. S16. The results are plotted for $K_{I}$ against the far-field stress, $\sigma_{x\infty}$, in Figure S9c and demonstrate the linear increase in $K_{I}$ with the increase in far-field stress, $\sigma_{x\infty}$. However, according to Griffth’s energy balance approach, the condition for crack initiation is for the stress intensity factor ($K_{I}$) to reach the critical value or the fracture toughness ($K_{IC}$).^[27]^

$K_{IC}$ of the cement paste obtained from the SENB experiments is plotted in Figure S10c (as shown by the gray line). The intersection of $K_{I}$ vs $\sigma_{x\infty}$ plot with the critical stress intensity factor, $K_{IC}$, yields the far-field stress at failure, $\sigma_{x\infty, F}$, as illustrated in Figure S9c for solid, and tubular Circular, 40%, and Elliptical, 40% *e = 2.5* cases. It is found that for a single tube ahead of the crack tip, the far-field stresses at failure for tubular, $\sigma_{x\infty,F}^{Tubular (circular or elipitical)}$, are both smaller than that of a monolithic solid, $\sigma_{x\infty,F}^{Solid}$, as shown in Figure S9c.^[24]^ This indicates that the required stress for initiation of the crack from the notch tip is lower in the case where tubes are present compared to the solid case without any tube ahead of the tip. Thus, $F\left( y_{1},y_{2} \right)>1$ when a tube is present ahead of the crack tip.

Therefore, by equating the Eq. S15 and Eq. S16 to $K_{IC}$ and using Eq. S26, the relation between the far-field stresses at failure for tubular material, $\sigma_{x\infty,F}^{Tubular (circular or elipitical)}$, and solid, $\sigma_{x\infty,F}^{Solid}$, can be written as Eq. S27.

$$\sigma_{x\infty,F}^{Tubular}=\frac{\sigma_{x\infty,F}^{Solid}}{F\left( y_{1},y_{2} \right)} (Eq. S27)$$

Assuming that the far-field stresses are directly proportional to the applied load in linear elastic material, the load at failure for tubular material, $P_{F}^{Tubular}$, and solid, $P_{F}^{Solid}$, can be illustrated by Eq. S28.

$$P_{F}^{Tubular}=\frac{P_{F}^{Solid}}{F\left( y_{1},y_{2} \right)} (Eq. S28)$$

And the normalized load at failure, $P_{F,N}$, of tubular architected material with respect to solid can be defined using the Eq. S29.

$$P_{F,N}= \frac{P_{F}^{Tubular}}{P_{F}^{Solid}}=\frac{1}{F\left( y_{1},y_{2} \right)} (Eq. S29)$$

The stress intensity factor ratio, $F\left( y_{1},y_{2} \right)=K_{I,Tubular}/K_{I,Solid}$, for different tubes is calculated using Eq. S26 and the results are presented in Figure S9d. It is found that $F\left( y_{1},y_{2} \right)$ increases with the increasing porosity (diameter) in the circular tube and decreases with the increasing aspect ratio in elliptical tubes. This indicates that the stress intensity factor can increase by as high as a factor of 1.5 due to the presence of a tube in front of the crack tip (Figure S9d).

Therefore, based on Eq. S29, the normalized load at failure, $P_{F,N}$, follows the opposite trend where it decreases with the increasing porosity (diameter) of the tube and increases with increasing aspect ratio as illustrated in Figure S9e. This theoretical trend is intuitive and indicates that the peak load decreases by a factor of 0.75 due to the addition/presence of the tube.

These trends of the theoretical $P_{F,N}$, in relation to porosity and aspect ratio, correspond closely with the trends of experimental $P_{F,N}$ derived from the SENB test on both tubular architected and solid materials, as shown in Figure S9f. However, there is a noticeable difference between the experimental and theoretical values of $P_{F,N}$ and the theoretical at the lower bounds which has to do with the superposition of other tubes in the experiments. In the theoretical analysis of the stress intensity factor, only the effect of a single tube on the stress intensity factor is considered whereas experimentally the stress intensity factor at the crack tip is also influenced by the presence of additional tubes in the architected material.

**Figure. S9. Theoretical and experimental analysis of stress intensity factor.** a) Schematics of a pre-existing crack (notch) in the monolithic material and b) the interaction of hollow tube with the crack under far-field tensile stresses, c) Stress intensity factor (K_I_) vs. far-field stress at failure for solid, Circular, 40%, and Elliptical, 40% *e = 2.5* along with the critical stress intensity factor of cement paste, d) Theoretical Mode-I stress intensity factor (SIF) ratio of tubular materials vs. solid counterpart obtained from Eq. S26, e) Theoretical and f) experimental normalized load at failure of architected materials vs. solid counterpart.

**Effect of the Shape and Location of the Tube on Mode-I Stress Intensity Factor in Tubular Architected Materials**

In considering the superposition of the tube, the location and the shape of the tube relative to the orientation of the crack (notch) presents additional alteration to the stress intensity factor at the notch tip. **Figure S10** illustrates how the normalized Mode-I stress intensity factor, $F\left( y_{1},y_{2} \right)$, varies depending on the tube's location along the x-axis, $x_{o}$, in relation to the crack tip for Circular, 40% and Elliptical, 40% *e* = 2.5.

The influence of the circular or elliptical tubes on the Mode-I stress intensity factor ratio, $F\left( y_{1},y_{2} \right),$ of the crack tip is most pronounced when the tube is directly in front of the crack tip, specifically when $x_{o}$ = 0 (Figure S9). As the tube begins to move away from the crack tip, as indicated by an increase in $x_{o}$, the stress intensity factor ratio rapidly decreases. When the circular or elliptical center point of the tube is located at $x_{o}$ of 7.0 mm or 9.0 mm respectively, the stress intensity factor ratio is equal to 1, indicating no effect on the stress intensity factor ratio.

Furthermore, as the distance of the tube from the notch continues to increase, the Mode-I stress intensity factor ratio drops below 1. Considering the inverse relation between load at failure and stress intensity factor ratio based on Eq. S29, the presence of a tube at a distance (larger than 7.0 and 9.0 mm) amplifies the load required for failure at the crack tip. However, at greater distances (larger than ~ 60 mm), the normalized stress intensity factor reverts to approach the value of 1, which signifies the diminishing effect of the presence of a tube on the stress intensity factor at the notch. Therefore, if the superposition of the tubes is to be considered, the competing effects will be in play on the required load at failure, especially between the tubes within or beyond the stress intensity factor ratio of 1. The theoretical determination of the stress intensity factor can be extended for comparison with experiments considering both superimposing the effect of multiple tubes at various locations ahead of the crack as well as the bending stress state.

**Figure S10.** a) A single tube located at the offset of $x_{o}$ with respect to crack tip, b) Theoretical stress intensity factor ratio, $F\left( y_{1},y_{2} \right),$ of notch tip with respect to offset, $x_{o}$, for circular and elliptical tube obtained from Eq. S26.

It is important to note that the increase in the aspect ratio of the tube (ellipticity) leads to decrease in the Mode-I stress intensity factor, which in turn increases the load-at-failure with increasing aspect ratio (*e)* as shown in Figure S9 d-f and **Figure S11a, c***.* The increase in load-at-failure subsequently lead to higher resistance to crack initiation, hence, resulting in an increase in the crack initiation fracture toughness, K_IC_, of the tubular architected materials with increasing aspect ratio.

The orientation of the tube can principle also alter the stress intensity factor, K_IC_. Positioning elliptical tube along their longer axis, as shown in Figure S11b, which involves rotating the tubes by 90 degrees, orients the more highly curved end of the elliptical tube toward the crack. This orientation increases the mode-I theoretical stress intensity factor (SIF) by 16 – 20% compared to when elliptical tube is aligned along the shorter axis, as illustrated in Figure S11c. This increase in stress intensity factor reduces the load-at-failure and, consequently, lowers the crack initiation fracture toughness (K_IC_) when tubes are oriented along their longer axis.

Additionally, once the crack is propagation from the notch to the elliptical tube, a higher stress concentration factor is developed in the elliptical tubes that is aligned along the longer axis compared to those aligned along the shorter axis.^[26,28]^ In that case, in context of step-wise cracking for crack reinitiation from the tube (and subsequent tubes), we can hypothesize that higher stress concentration in “along the longer axis” case, leads to a lower load-at-failure compared to the “along the shorter axis” case, thus could negatively impact the stepwise cracking mechanism. In other words, a circular design is hypothesized to outperform the “along longer axis” design and underperform the “along the longer axis” design in terms of step-wise cracking from the tube.

**
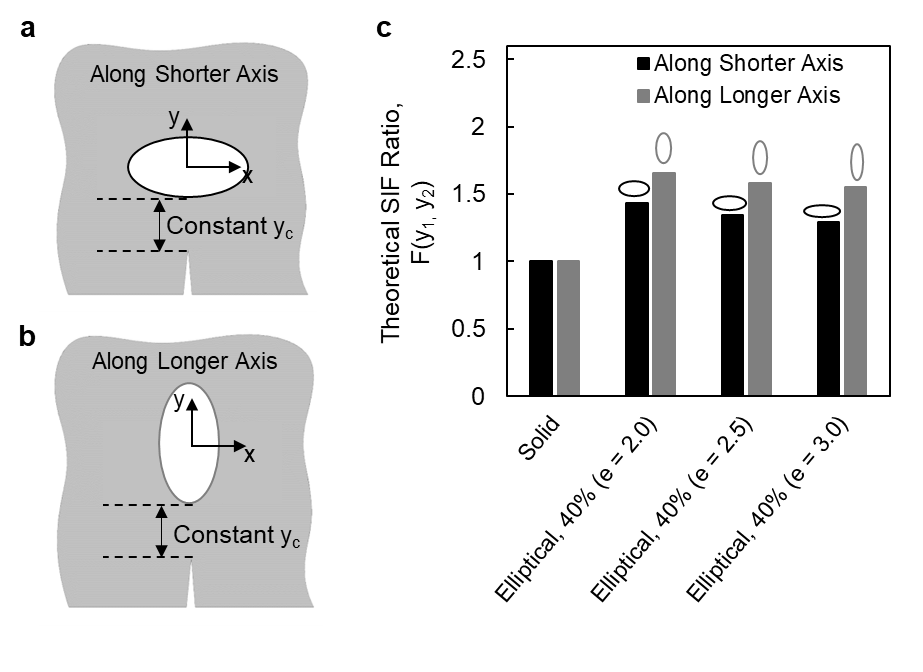
**

**Figure S11.** **A single elliptical tube positioned ahead of the crack in two orientations, leading to variation on theoretical stress intensity factor**: a) with the shorter axis aligned with the crack and b) with the longer axis aligned with the crack. In both scenarios, the distance between the crack tip and the front of the tube, labeled as y_c_, is maintained constant for a specific aspect ratio, c) Theoretical stress intensity factor ratio, $F\left( y_{1},y_{2} \right),$ of the notch tip for elliptical tubes with different aspect ratios for two different orientations.

**Movies**.

Movie S1. Digital Image Correlation (DIC) of the SENB testing of Circular architected materials with 40% porosity.

Link: <https://youtu.be/qIQi26O5RP8>

Movie S2. Digital Image Correlation (DIC) of the SENB testing of Elliptical architected materials with 40% porosity and aspect ratio, e = 2.5.

Link: <https://youtu.be/JlM-xXBLfUE>

**References**

[1] ASTM E1820-20b, *ASTM International* **2022**, *03*.

[2] H. Wan, N. Leung, U. Jargalsaikhan, E. Ho, C. Wang, Q. Liu, H.-X. Peng, B. Su, T. Sui, *Mater Des* **2022**, *223*, 111190.

[3] V. Naglieri, B. Gludovatz, A. P. Tomsia, R. O. Ritchie, *Acta Mater* **2015**, *98*, 141.

[4] T. Li, Y. Chen, L. Wang, *Compos Sci Technol* **2018**, *167*, 251.

[5] Z. P. Bazant, J. Planas, *Fracture and Size Effect in Concrete and Other Quasibrittle Materials*, Routledge, **2019**.

[6] Y. Jenq, S. P. Shah, *J Eng Mech* **1985**, *111*, 1227.

[7] H. Tada, P. C. Paris, G. R. Irwin, *Handbook, Del Research Corporation* **1973**, *34*.

[8] J. E. Srawley, in *Conf. of Am. Soc. for Testing and Mater., Committee E-24*, **1976**.

[9] H.-L. Gao, S.-M. Chen, L.-B. Mao, Z.-Q. Song, H.-B. Yao, H. Cölfen, X.-S. Luo, F. Zhang, Z. Pan, Y.-F. Meng, *Nat Commun* **2017**, *8*, 287.

[10] P. Albrecht, W. R. Andrews, J. P. Gudas, J. A. Joyce, F. J. Loss, D. E. McCabe, D. W. Schmidt, W. A. VanDerSluys, *J Test Eval* **1982**, *10*, 245.

[11] D. D. Higgins, J. E. Bailey, *J Mater Sci* **1976**, *11*, 1995.

[12] R. Gettu, Z. P. Bazant, M. E. Karr, *ACI Mater J* **1990**, *87*, 608.

[13] B. Gross, J. E. Srawley, *SPIE MILESTONE SERIES MS* **1997**, *138*, 256.

[14] A. Sidorova, E. Vazquez-Ramonich, M. Barra-Bizinotto, J. J. Roa-Rovira, E. Jimenez-Pique, *Constr Build Mater* **2014**, *68*, 677.

[15] L. J. Parrott, *Magazine of Concrete Research* **1974**, *26*, 198.

[16] C.-J. Haecker, E. J. Garboczi, J. W. Bullard, R. B. Bohn, Z. Sun, S. P. Shah, T. Voigt, *Cem Concr Res* **2005**, *35*, 1948.

[17] ASTM C293/C293M-16, *ASTM International* **2013**, *4*.

[18] S. Torquato, H. W. Haslach Jr, *Appl. Mech. Rev.* **2002**, *55*, B62.

[19] N. Kapernaum, F. Giesselmann, *Phys Rev E* **2008**, *78*, 062701.

[20] T. M. Truskett, S. Torquato, P. G. Debenedetti, *Phys Rev E* **2000**, *62*, 993.

[21] S. Torquato, T. M. Truskett, P. G. Debenedetti, *Phys Rev Lett* **2000**, *84*, 2064.

[22] H. Jelitto, G. A. Schneider, *Acta Mater* **2018**, *151*, 443.

[23] P. Albrecht, K. Yamada, *Journal of the Structural Division* **1977**, *103*, 377.

[24] J. Tirosh, A. S. Tetelman, *Int J Fract* **1976**, *12*, 187.

[25] W. Yi, Q. Rao, W. Zhu, Q. Shen, Z. Li, W. Ma, *Adv. Appl. Math. Mech* **2022**, *14*, 125.

[26] X.-L. Gao, *International Journal of Pressure Vessels and Piping* **1996**, *67*, 95.

[27] M. Janssen, J. Zuidema, R. Wanhill, *Fracture Mechanics: Fundamentals and Applications*, CRC Press, **2004**.

[28] A. Lu, N. Zhang, G. Zeng, *Acta Mechanica Solida Sinica* **2017**, *30*, 318.
